# The diagnostic performance of the ductus venosus for the detection of cardiac defects in the first trimester: a systematic review and diagnostic test accuracy meta-analysis

**DOI:** 10.1007/s00404-022-06812-w

**Published:** 2022-10-31

**Authors:** Fabiana Savoia, Carolina Scala, Marlene Coppola, Gaetano Riemma, Salvatore Giovanni Vitale, Mislav Mikuš, Antonio Schiattarella, Marco La Verde, Nicola Colacurci, Pasquale De Franciscis, Maddalena Morlando

**Affiliations:** 1 Department of Woman, Child and General and Specialized Surgery, Obstetrics and Gynecology Unit, University of Campania “Luigi Vanvitelli”, Naples, Italy; 2 Division of Obstetrics and Gynecology, Gaslini Institute, Genoa, Italy; 3 Obstetrics and Gynecology Unit, Department of General Surgery and Medical Surgical Specialties, University of Catania, Via Santa Sofia 78, 95123 Catania, Italy; 4 Department of Obstetrics and Gynecology, University Hospital Center Zagreb, Zagreb, Croatia

**Keywords:** Ductus venosus, Congenital heart defects, Diagnostic accuracy, First trimester screening, Ultrasonography

## Abstract

**Purpose:**

Abnormal flow in the ductus venosus (DV) has been reported to be associated with adverse perinatal outcome, chromosomal abnormalities, and congenital heart defects (CHD). Aneuploid fetuses have increased risk of CHD, but there are discrepancies on the performance of this markers in euploid fetuses. The aim of this meta-analysis was to establish the predictive accuracy of DV for CHD.

**Methods:**

MEDLINE, EMBASE, and CINAHL were searched from inception to February 2022. No language or geographical restrictions were applied. Inclusion criteria regarded observational and randomized studies concerning first-trimester DV flow as CHD marker. Random effect meta-analyses to calculate risk ratio (RR) with 95% confidence interval (CI), hierarchical summary receiver-operating characteristics (HSROC), and bivariate models to evaluate diagnostic accuracy were used. Primary outcome was the diagnostic performance of DV in detecting prenatal CHD by means of area under the curve (AUROC). Subgroup analysis for euploid, high-risk, and normal NT fetuses was performed. Quality assessment of included papers was performed using QUADAS-2.

**Results:**

Twenty two studies, with a total of 204.829 fetuses undergoing first trimester scan with DV Doppler evaluation, fulfilled the inclusion criteria for this systematic review. Overall, abnormal DV flow at the time of first trimester screening was associated to an increased risk of CHD (RR 6.9, 95% CI 3.7–12.6; *I*^2^ = 95.2%) as well in unselected (RR: 6.4, 95% CI 2.5–16.4; *I*^2^ = 93.3%) and in euploid (RR: 6.45, 95% CI 3.3–12.6; *I*^2^ = 95.8%) fetuses. The overall diagnostic accuracy of abnormal DV in detecting CHD was good in euploid fetuses with an AUROC of 0.81 (95% CI 0.78–0.84), but it was poor in the high-risk group with an AUROC of 0.66 (95% CI 0.62–0.70) and in the unselected population with an AUROC of 0.44 (95% CI 0.40–0.49).

**Conclusions:**

Abnormal DV in the first trimester increases the risk of CHD with a moderate sensitivity for euploid fetuses. In combination with other markers (NT, TV regurgitation) could be helpful to identify fetuses otherwise considered to be at low risk for CHD. In addition to the improvement of the fetal heart examination in the first trimester, this strategy can increase the detection of major CHD at earlier stage of pregnancy.

**Supplementary Information:**

The online version contains supplementary material available at 10.1007/s00404-022-06812-w.

## What does this study add to the clinical work

**Table:** 

Ductus venosus (DV) evaluation during first trimester ultrasound has been proposed to increase the detection rate of cardiac defects. This diagnostic test accuracy meta-analysis showed that DV has good diagnostic accuracy for cardiac defects in euploid fetuses but poor in high-risk or unselected populations.	

## Introduction

Congenital heart defects (CHD) are the most common malformations diagnosed during fetal life, representing around one-third of congenital abnormalities [1]. In addition, stillbirth and neonatal death may be complications of CHD in approximately 5 and 20% of cases, respectively [2, 3].

The screening for CHD is usually performed during the second trimester anomaly scan, with a detection rate up to 60% [4]. When a specialist performs the echocardiographic evaluation, the detection rate increases, even at lower gestational ages [5–7]. Despite this, a large proportion of CHD remains undetected at birth, leading to a poor prognosis, especially for the critical CHD, which would require urgent medical and surgical care at birth [8, 9]. Therefore, prenatal diagnosis is crucial to improve fetal outcome of fetuses with CHD [10, 11].

The well-known association between increased nuchal translucency (NT) during the first trimester screening scan and CHD [12–15] has led to improved first-trimester CHD detection rates, through an ultrasound evaluation of the fetal heart at this early stage. However, CHD diagnosis in the first trimester remains challenging, and the detection rates are very heterogeneous when considering different studies [16], ranging from 34% in the largest study performed on 45.000 pregnancies [17] to 10% or less [18, 19].

The use of additional parameters, such as NT and Doppler examination of ductus venosus and tricuspid valve, has been proposed to achieve a higher detection rate of CHD during the first trimester [20].

Increased NT at 11–14 weeks in euploid fetuses has been proven to be an early marker of CHD [21]. In addition, a recent meta-analysis showed that a NT above the 99th centile (i.e. 3.5 mm) can identify around 30% of fetuses with CHD, and it can be considered the strongest predictor of CHD in the first trimester [22].

Tricuspid regurgitation seems to correlate well with CHD in high-risk fetuses (i.e. those with increased NT) but not in low risk ones [23].

The finding of an abnormal ductus venosus (DV) in the first trimester increases the risk of adverse perinatal outcome, such as chromosomal anomalies and CHD [24, 25]. A systematic review published in 2011 demonstrated that the DV waveform examination has a moderate sensitivity, around 50%, for the detection of CHD, and it is even higher (83%) in fetuses with increased NT, while in approximately 96% of fetuses with normal NT and no CHD, DV waveform is normal [26]. The purpose of this systematic review, which represents an update of one published in 2011, is to establish the diagnostic performance of DV for the detection of CHD in the first trimester scan. The secondary aim is to study the strength of this association.

## Materials and methods

### Search strategy

We performed this quantitative analysis according to an a-priori-designed protocol recommended for systematic reviews and meta-analyses [27]. MEDLINE, EMBASE, and CINAHL were searched electronically since inception on 20 December 2019 and updated on 01 February 2022. A combination of the following relevant medical subject heading (MeSH) terms, keywords and word variants was used: “fetus”, “ductus venosus”, “first trimester”, and “CHD”. Searches were also performed on PsycINFO and AMED to find other relevant papers and reduce publication bias. Moreover, to search for abstracts of international and national conferences, the grey literature (NTIS, PsycEXTRA) was also screened. Reference lists of relevant articles and reviews were hand-searched for additional reports. No language or geographic location restriction was applied. Commentaries, letters to the editor, editorials, and reviews were excluded from the search.

The analysis was carried out according to the Preferred Reporting Items for Systematic Reviews and Meta-Analysis (PRISMA) [28] and the Synthesizing Evidence from Diagnostic Accuracy TEsts (SEDATE) guidelines [29]. The study was registered with the PROSPERO database (CRD42020163214).

### Study selection, data collection and data items

The studies were first screened for eligibility considering the titles and abstracts, and the final decision for inclusion was based on the evaluation of the full-text articles. Studies were included when they provided data on the presence or not of CHD according to the DV waveform in the first trimester. The methodology of the included studies was evaluated to rule out the following potential biases: characteristics of the population, prevalence of CHD in the population, ultrasound methodology and gestational age at ultrasound. The primary outcome of this systematic review was to establish the diagnostic performance of DV for the prenatal detection of CHD in the first trimester scan; the secondary aim was to study the strength of this association. As outlined in each one of the included studies, these outcomes were evaluated in overall synthesis of the available population of fetuses undergoing first trimester screening and subsequently stratified as follows:

- Unselected general population: studies in which the risk for CHD was not stratified according to maternal or fetal causes, including NT and maternal age below thresholds (NT above 95th or 99th centile and maternal age over 40 years).
- Euploid fetuses: fetuses with a normal euploid karyotype.
- High-risk fetuses: fetuses with increased risk for CHD, including increased NT above 95th or 99th centile, first trimester regurgitation or reversed/absent DV a-wave, maternal diabetes, previous child with CHD, use of anticonvulsant therapy.
- Normal NT fetuses: fetuses with NT below the 95th or 99th centile.

For the ultrasound methodology, only studies with an appropriate and precise description of the technique were included. The technique, according to the guidelines of The Fetal Medicine Foundation (FMF), has to fulfill the following criteria: (a) operators performed the examinations during fetal quiescence; (b) the magnification of the image was such that the fetal thorax and abdomen occupied the whole screen; (c) a right ventral mid-sagittal view of the fetal trunk was obtained and color-flow mapping was used to demonstrate the umbilical vein, ductus venosus and fetal heart; (d) the pulsed Doppler sample was small (0.5–1.0 mm) to avoid contamination from the adjacent veins and it was placed in the yellowish aliasing area, which is the portion immediately above the umbilical sinus; (e) the insonation angle was less than 30◦; (f) the filter was set at a low frequency (50–70 Hz) to allow visualization of the whole waveform; and (g) the sweep speed was high (2–3 cm/s) so that the waveforms were widely spread, allowing better assessment of the A-wave. Waveforms were assessed qualitatively and considered to be abnormal if the A-wave was absent or reversed.

Two authors (F.S. and M.C.) reviewed all abstracts independently. Agreement regarding potential relevance was reached by consensus; full-text copies of those papers were obtained and the same two reviewers independently extracted relevant data regarding the number of pregnancies, gestational age at ultrasound, karyotype (when specified), and prevalence of abnormal DV blood flow in fetuses with and without CHD. In case of inconsistencies, the reviewers discuss the paper to reach a consensus, and if necessary, request discussion with a third author (M.M.). If more than one study was published on the same cohort with identical endpoints, the report containing the most comprehensive information on the population was included to avoid overlapping populations. For those articles in which information was not reported, but the methodology was such to suggest that this information would have been recorded initially, the authors were contacted. Case reports, conference abstracts, and case series with fewer than three cases were excluded to avoid publication bias.

### Statistical analysis

Random effect meta-analysis was used to compare subjects with abnormal DV versus normal DV in predicting CHD in euploid fetuses. Only studies reporting a direct assessment of the risk were meta-analyzed. The results were reported as relative risks (RR) for the outcome observed.

For each study included, we extract the following statistical parameters: true positive (TP), true negative (TN), false positive (FP) and false negative (FN), when not available, we calculated them and arranged a 2 × 2 table using the formula: sensitivity = TP/(TP + FN) and specificity = TN/(FP + TN). Both hierarchical summary receiver-operating characteristics (HSROC) [30] and bivariate models were used. To evaluate the overall predictive accuracy of abnormal DV blood flow for the detection of CHD, we calculate the likelihood ratio (LR) and diagnostic odds-ratio (DOR) using the random-effect model by DerSimonian-Laird and the area under the curve (AUROC) using the bivariate model by Reitsma. The AUROC was interpreted in a four-grade scale as follows: AUROC < 0.75 not accurate, 0.75–0.92 good, 0.93–0.96 very good, AUROC > 0.97 excellent. The DOR was defined as the ratio of the odds of the test being positive if the subject has a disease, relative to the odds of the test being positive if the subject does not have the disease, i.e. LR + /LR– [31]. A LR +  < 2 was recognized as not meaningful, between 2 and 5, between 5 and 10 with a small and moderate, and > 10 were recognized as a not meaningful, small, moderate, and large increase in probability, respectively. LR− in the range of > 0.5, between 0.2 and 0.5, between 0.1 and 0.2, and < 0.1 were interpreted as a not meaningful, small, moderate, and large decrease of probability. For each study, the prevalence of CHD was calculated in the overall population, in the euploid and in the high-risk populations. Heterogeneity was assessed using the Higgins *I*^2^ index in which 0% means no heterogeneity and 100% represents the highest degree of heterogeneity [32]. Publication bias was assessed using the Deek funnel plot asymmetry test for each outcome and each subgroup, and a *p* value < 0.05 was considered to reflect significant publication bias. Stata 14.1 (StataCorp LLC, College Station, TX) and Review Manager 5.3 (The Nordic Cochrane Centre 2014, Copenhagen, Denmark) were used for all data analyses.

### Risk of bias

The methodology of the included studies was analyzed by three authors (F.S, M.C. and C.S.) by means of the qualitative instrument for data collection (Quality Assessment of Diagnostic Accuracy Studies-2; QUADAS-2), as recommended by the Healthcare Research and Quality Agency [17]. This tool is made up of four domains including (a) patient selection, (b) index test, (c) reference standard, and (d) flow and timing. All domains are assessed for risk of bias and the first three domains are assessed for applicability by indicating a low, high, or unclear risk. Publication bias among the included studies was evaluated by means of the Deek’s funnel plot test.

## Results

**Fig. 1:**
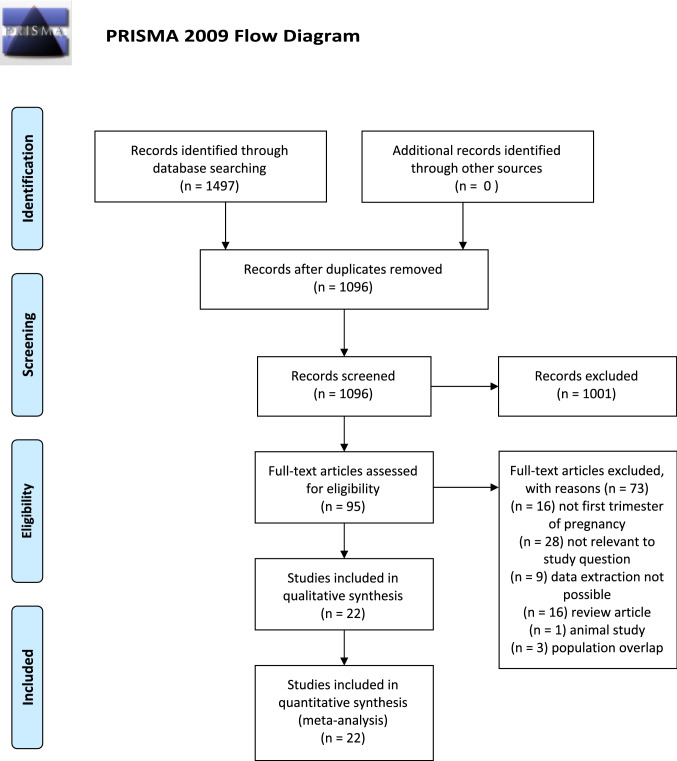
PRISMA flow diagram of included studies

A total of 1096 articles were identified and assessed with respect to their eligibility for inclusion. Of those, 95 had the full text assessed for eligibility and 22 fulfilled the inclusion criteria for this systematic review (Fig. 1). Supplementary Table 1 lists the excluded studies and the reason for exclusion. Table 1 lists the general characteristics of the included studies. The twenty-two studies included were all prospective, except for two retrospective analyses, with a total of 204.829 fetuses undergoing first trimester scan with DV Doppler evaluation (Table 1).

**Table 1:** Main characteristics of studies included in quantitative synthesis

Author	Country	Design	Duration	Sample size	Population	NT Cut-off	DV Cut-off	CHD prevalence (%)
Wagner	Germany	Retrospective	2010–2017	528	Euploid	> 99 centile	Absent or reversed flow during A-wave	48 (9.1)
Wiechec	Poland	Retrospective	2009–2012	5816	General	> 95 centile	Absent or reversed flow during A-wave	28 (0.4)
Burger	Netherlands–Portugal	Prospective	NA	5	General	> 95 centile	Absent or reversed flow during A-wave	3 (60.0)
Mula	Spain	Prospective	2012–2014	418	General	> 95 centile	Absent or reversed flow during A-wave	27 (6.4)
Turan	USA	Prospective	2007–2012	164	High risk	> 95 centile	Absent or reversed flow during A-wave	20 (12.2)
Yang	China	Prospective	NA	4673	General	> 95 centile	Absent or reversed flow during A-wave	31 (0.6)
Borrell	Spain	Prospective	2002–2009	12,401	General	> 95 centile	Absent or reversed flow during A-wave	36 (0.2)
Prats	Spain	Prospective	2003–2009	9483	General	> 95 centile	Absent or reversed flow during A-wave	48 (0.5)
Volpe	Italy	Prospective	2009–2010	2976	General	> 95 centile	Absent or reversed flow during A-wave	28 (0.9)
Chelemen	United Kingdom	Prospective	2006–2009	40,990	Euploid	> 95 centile	Absent or reversed flow during A-wave	85 (0.2)
Clur	Netherlands	Prospective	2003–2009	27	High risk	> 95 centile	Absent or reversed flow during A-wave	8 (29.6)
Timmerman	Netherlands	Prospective	1996–2008	792	Euploid	> 95 centile	Absent or reversed flow during A-wave PI > 95 centile	26 (3.2)
Martinez	Spain	Prospective	2005–2009	5864	Euploid	> 99 centile	Absent or reversed flow during A-wave	45 (0.7)
Maiz	United Kingdom	Prospective	NA	191	Euploid	> 95 centile	Absent or reversed flow during A-wave	16 (8.4)
Maiz (2)	United Kingdom	Prospective	NA	10,490	General	> 95 centile	Absent or reversed flow during A-wave	20 (0.1)
Toyama	Brazil	Prospective	1998–2001	1097	General	> 95 centile	Absent or reversed flow during A-wave	7 (0.6)
Favre	France	Prospective	1999–2000	998	Euploid	> 95 centile	Absent or reversed flow during A-wave	10 (1.0)
Matias	Portugal	Prospective	NA	446	Euploid	> 95 centile	Absent or reversed flow during A-wave	7 (1.5)
Karadzov-Orlic	Serbia	Prospective	2006–2014	13,593	Euploid	> 95 centile	Absent or reversed flow during A-wave	116 (0.8)
Zoppi	Italy	Prospective	1999–2002	325	General	> 95 centile	Absent or reversed flow during A-wave	0 (0)
Minella	United Kingdom	Retrospective	2009–2018	93,209	Euploid	> 95 centile	Absent or reversed flow during A-wave	211 (0.2)
Murta	Brazil	Prospective	1998–2000	343	Euploid	> 95 centile	Absent or reversed flow during A-wave	0 (0)

General: unselected general population, *NA* not available, *NT* nuchal translucency, *DV* ductus venosus, *CHD* congenital heart defects, *PI* pulsatility index

Of the twenty-two studies included, ten were carried out in an unselected population, ten in an euploid population and two in a high-risk population for CHD. Among the ten studies performed in an euploid population, eight studies analyzed only chromosomally normal fetuses [33–42]. Of the two studies carried out in a high-risk population, the study by Clur S.A.B et al. included women referred for fetal echocardiography with known risk factors for CHD, such as: increased NT, an increased a-priori risk for CHD or suspicion of a CHD at ultrasound examination [43]. In the study by Turan S. et al., women were considered at high risk for CHD in case of: pre-gestational diabetes, in-vitro fertilization (IVF), increased NT, evidence of first-trimester tricuspid regurgitation (TR), reversed A-wave in the ductus venosus (DV), a previous child with CHD or anticonvulsant medications usage [44]. All the other ten studies included an unselected population of women undergoing first trimester screening [45–54]. Definition of abnormal DV blood flow was different among the included studies. In four studies the DV was abnormal when the PI was > 95th centile, in nine studies when the A-wave of the DV was either absent or reversed, and in eight studies when the A-wave was reversed. The methodological assessment of the included studies using the QUADAS-2 tool is shown in Supplementary table 2. The overall score for risk of bias and applicability concerns was low in the different categories.

### Data synthesis: overall

**Fig. 2:**
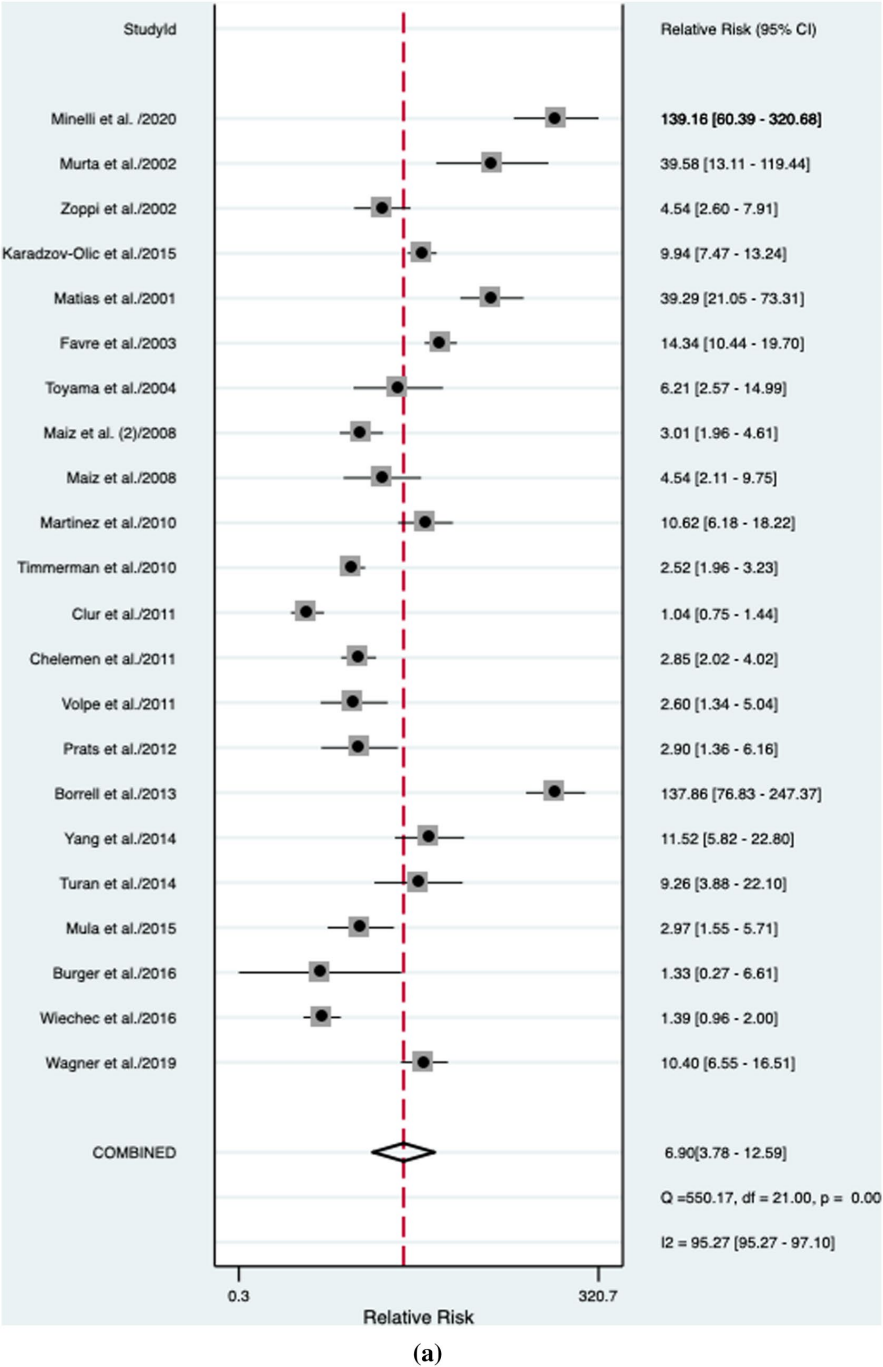

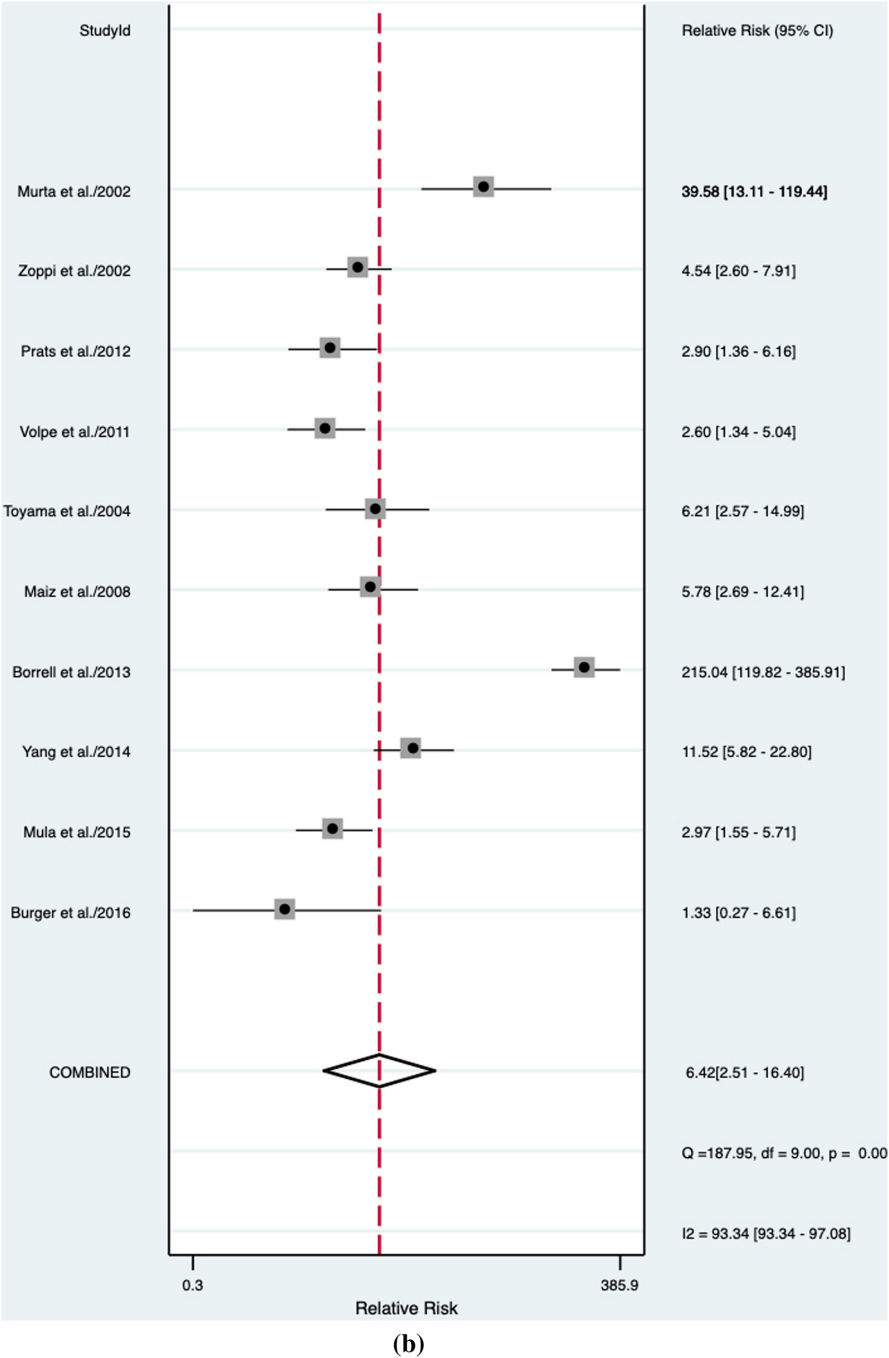

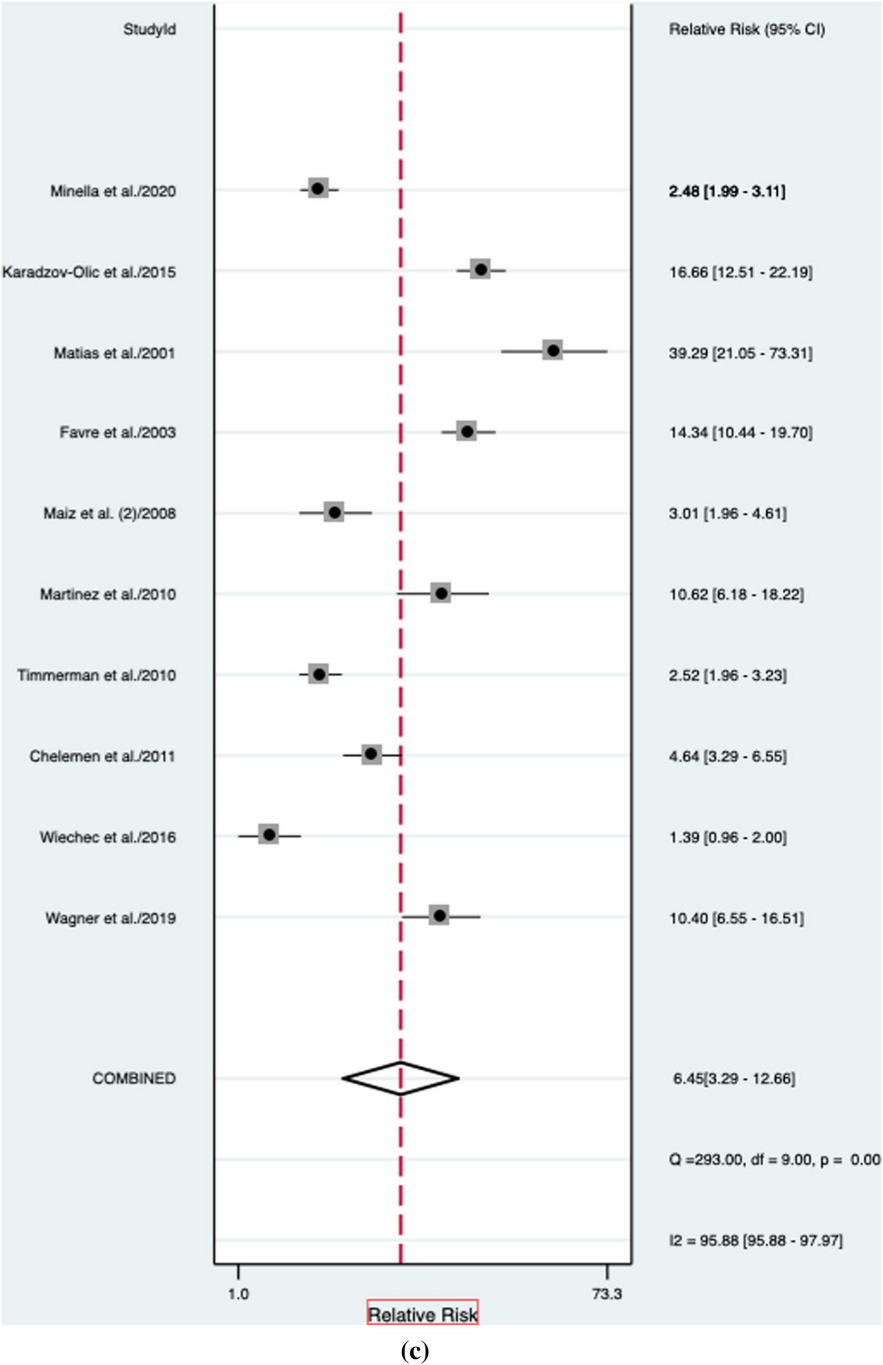

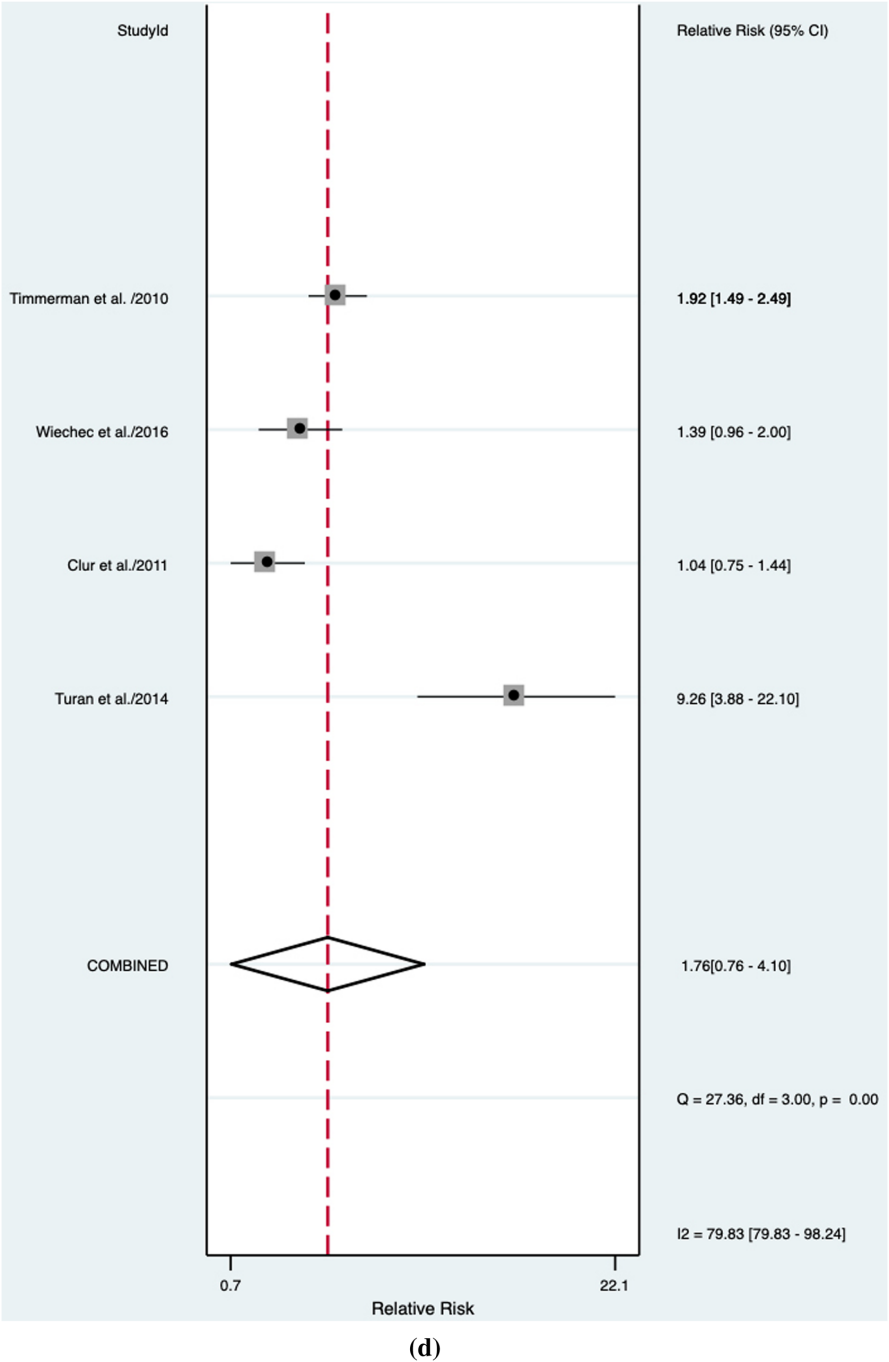

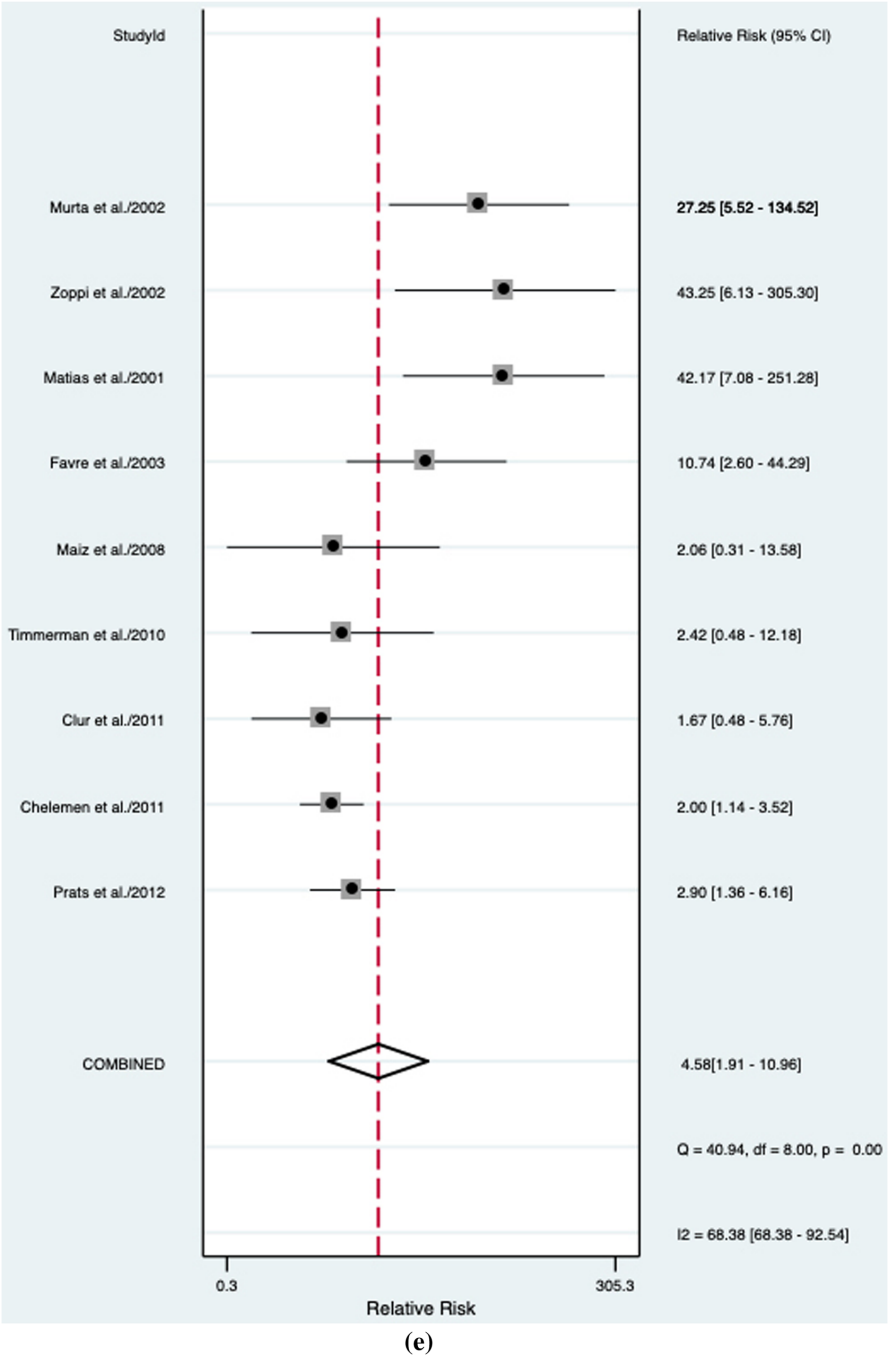
Association between abnormal ductus venosus flow and risk for congenital heart defects in **a** overall, **b** unselected, **c** euploid, **d** high-risk, and **e** normal NT fetuses. RR: relative risk

**Fig. 3:**
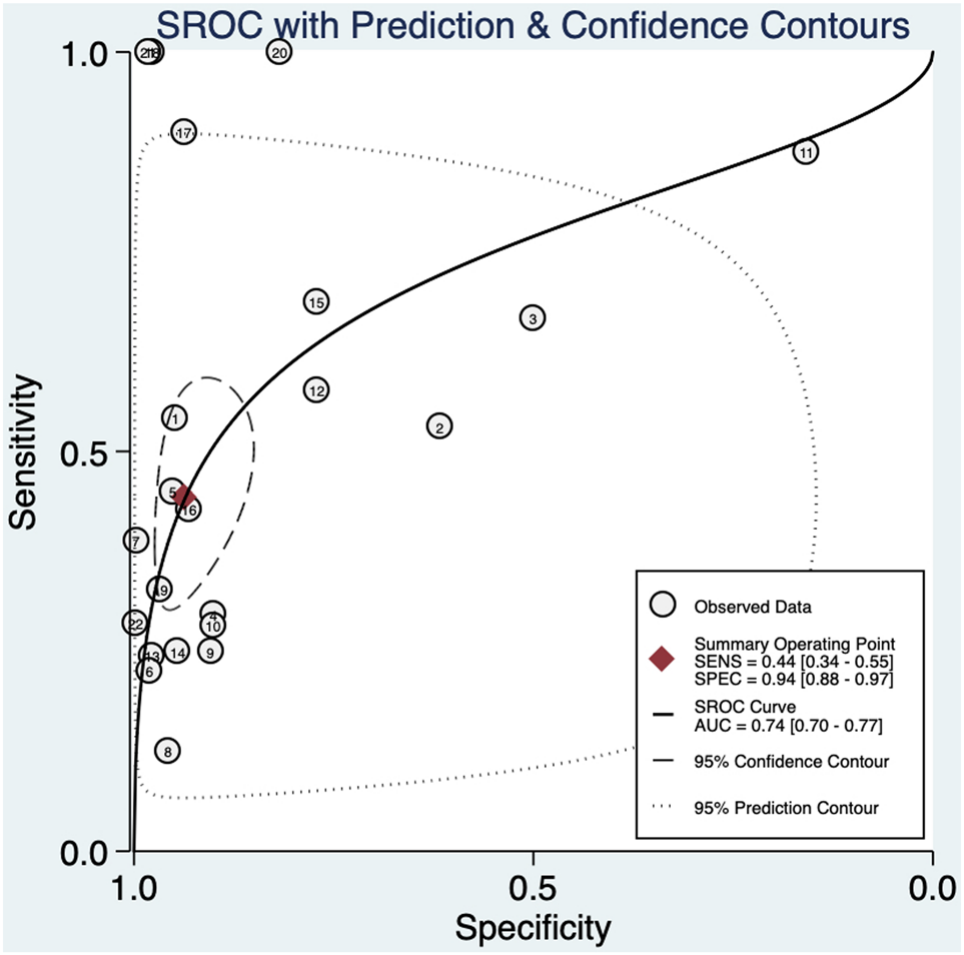
Hierarchical summary receiver operating characteristic curve for overall fetuses

An abnormal DV blood flow was associated to a higher risk of a CHD (RR: 6.9, 95% CI 3.7–12.6; *I*^2^ = 95.2%) (Fig. 2a). Abnormal DV blood flow had an overall poor diagnostic accuracy in detecting CHD with an AUROC of 0.74 [95% CI 0.70–0.77] (Fig. 3), a sensitivity of 44% [95% CI 0.34–0.55], a specificity of 94% [95% CI 0.88–0.97], a DOR of 12 [95% CI 6–22; *I*^2^ = 99%], a LR + of 6.9 [95% CI, 3.78–12.59], and LR–of 0.6 [95% CI, 0.50–0.72]. The Deek’s funnel plot asymmetry test revealed no publication bias for the overall analysis (*p* = 0.38) (Supplementary Fig. 1).

### Unselected general population

**Fig. 4:**
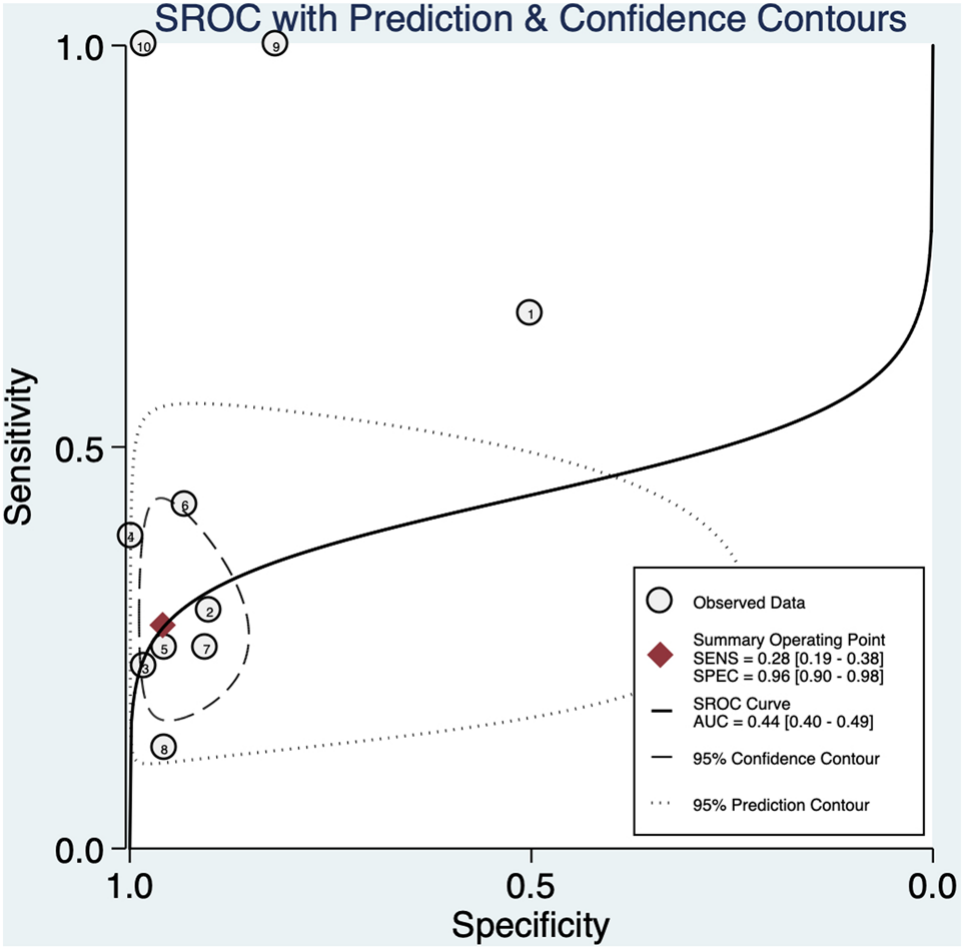
Hierarchical summary receiver operating characteristic curve for unselected fetuses

In the unselected general population, regardless to the fetal karyotype, an abnormal DV blood flow was associated with a significant higher risk of CHD (RR: 6.4, 95% CI 2.5–16.4; *I*^2^ = 93.3%) (Fig. 2b). Even though abnormal DV blood flow in this population [41–49] had a poor diagnostic accuracy for CHD detection with an AUROC of 0.44 [95% CI 0.40–0.49] (Fig. 4), a sensitivity of 28% [95% CI 0.19–0.38], a specificity of 96% [95% CI 0.90–0.98]., a DOR of 8 [95% CI 3–24; *I*^2^ = 96%], a LR + 6.4 [95% CI, 2.5–16.4], and LR–of 0.76 [95% CI, 0.66 – 0.87]. No publication bias was present at Deek’s funnel plot test (*p* = 0.93) (Supplementary Fig. 2).

### Euploid fetuses

**Fig. 5:**
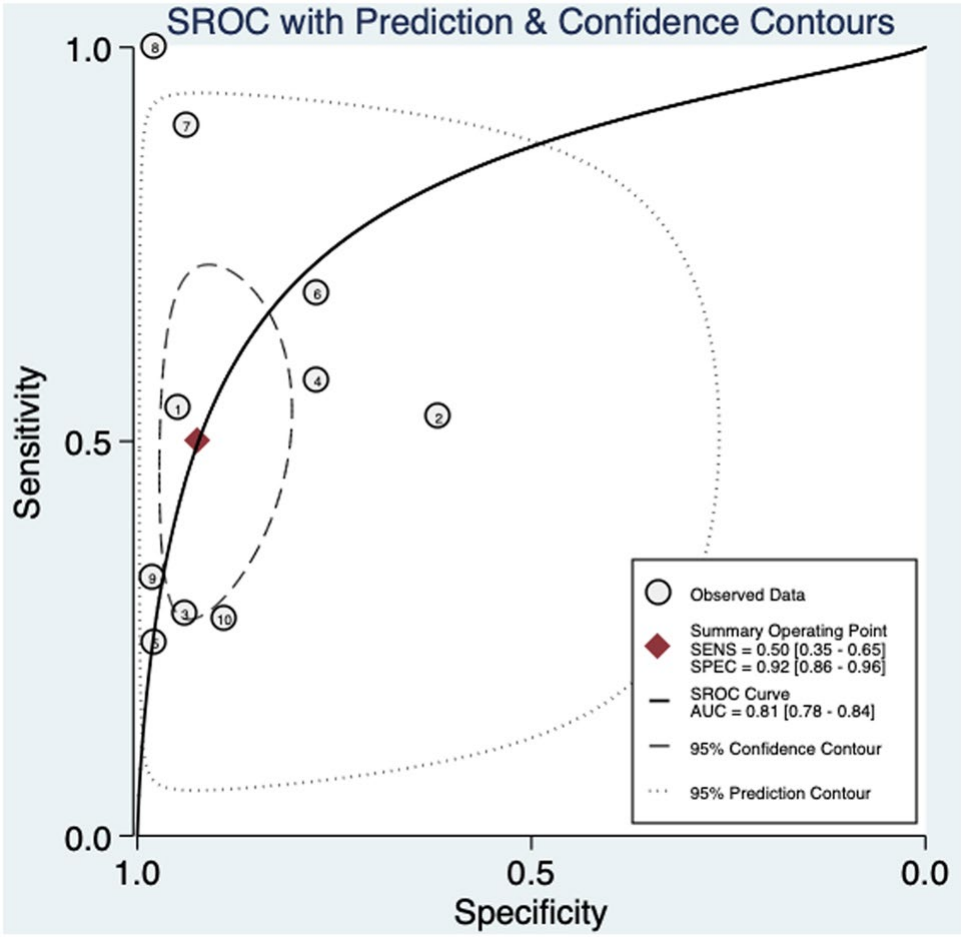
Hierarchical summary receiver operating characteristic curve for euploid fetuses

When considering euploid fetuses, an abnormal DV blood flow was still associated to a higher risk of CHD (RR: 6.45, 95% CI 3.3–12.6; I^2^ = 95.8%) (Fig. 2c). Abnormal DV blood flow in this population had a good diagnostic accuracy for the detection of CHD with an AUROC of 0.81 [95% CI 0.78–0.84] (Fig. 5), a sensitivity of 50% [95% CI 0.35–0.65], a specificity of 92% [95% CI 0.86–0.96], a DOR of 12 [95% CI 5–28; *I*^2^ = 99%], a LR + 6.4 [95% CI, 3.3–12.6], and LR–of 0.54 [95% CI, 0.40–0.74]. Deek’s asymmetry test revealed no publication bias in the above-mentioned subgroup of fetuses (*p* = 0.13) (Supplementary Fig. 3).

### High-risk fetuses

**Fig. 6:**
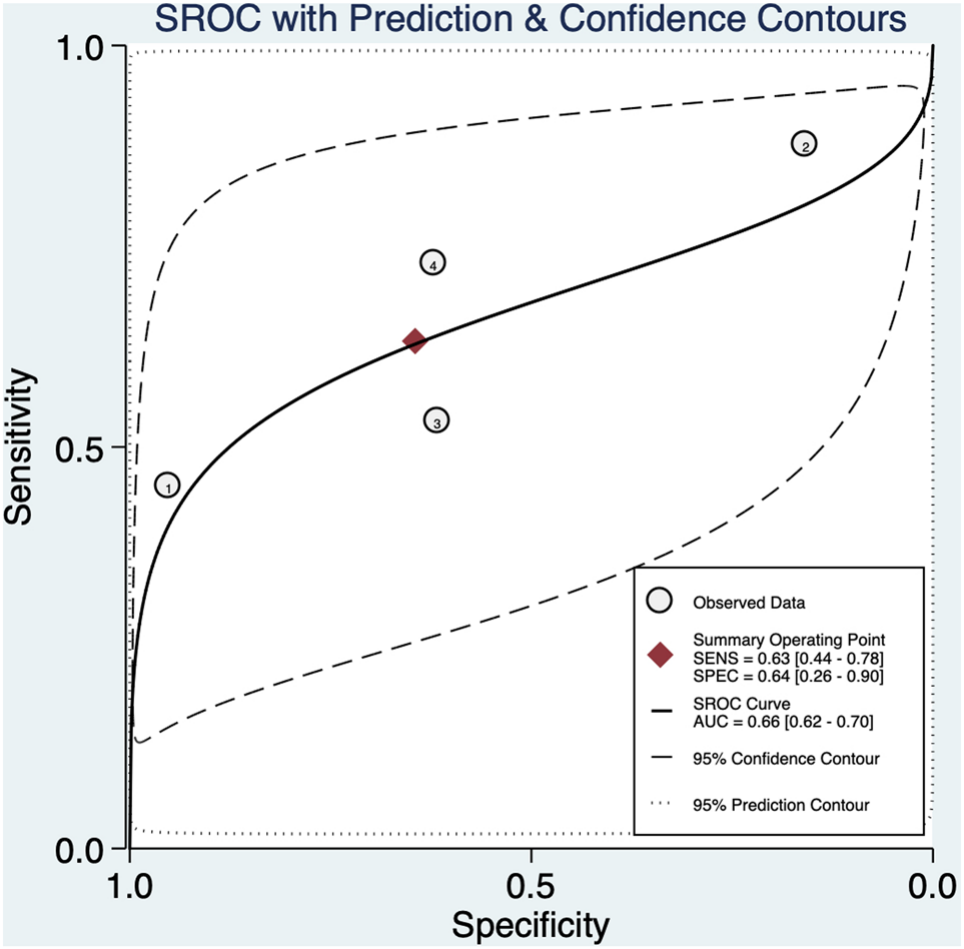
Hierarchical summary receiver operating characteristic curve for high-risk fetuses

The strength of the association between abnormal DV blood flow and CHD was not confirmed in the high-risk population (RR 1.8, 95% CI 0.8–4.1, *I*^2^ = 79.8%) (Fig. 2d). Abnormal DV blood flow in this population has a poor diagnostic accuracy for CHD detection with an AUROC of 0.66 [95% CI 0.62–0.70] (Fig. 6), a sensitivity of 63% [95% CI 0.44–0.78], a specificity of 64% [95% CI 0.26–0.90], *I*^2^ = 95%], a DOR of 3 [95% CI 1–10; *I*^2^ = 95%], a LR + 1.8 [95% CI, 0.8–4.1], and LR –0.58 [95% CI, 0.39–0.85]. Publication bias was not significantly present in Deek’s funnel plot (*p* = 0.97) (Supplementary Fig. 4).

### Normal NT fetuses

**Fig. 7:**
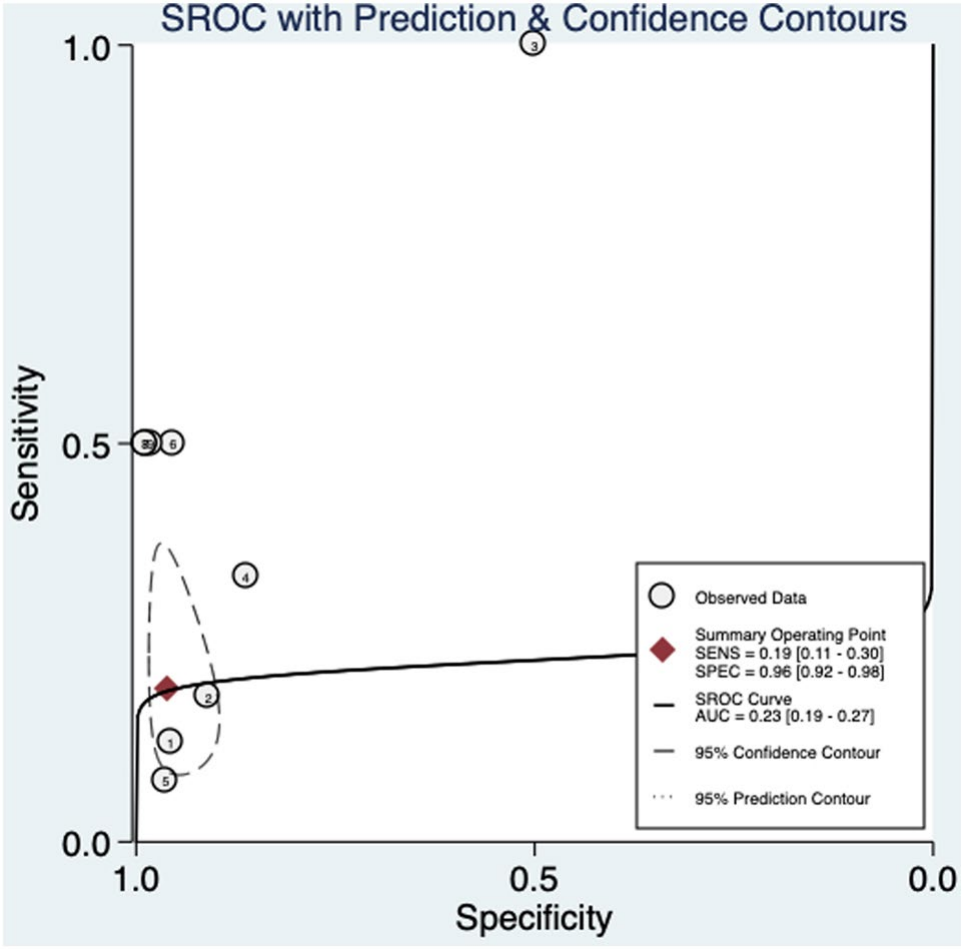
Hierarchical summary receiver operating characteristic curve for normal NT fetuses

For fetuses with a normal NT, an abnormal DV blood flow was related to an increased risk for CHD (RR 4.58, 95% CI 1.9–11.0, *I*2 = 68.4%) (Fig. 2e). In this sub-population, abnormal DV blood flow has a poor diagnostic accuracy in detecting CHD (AUROC 0.23 [95% CI 0.19–0.27] (Fig. 7), with a sensitivity of 19% [95% CI 0.11–0.30] and a specificity of 96% [95% CI 0.92–0.98], a DOR of 2 [95% CI 2–14] with LR + and LR− of 4.6 [95% CI 1.9 to 11.0] and 0.85 [95% CI 0.75 to 0.96], respectively. However, publication bias evaluated by means of Deek’s asymmetry test revealed potential small-study effect on these findings (*p* = 0.01) (Supplementary Fig. 5).

## Discussion

### Main findings

This systematic review showed that abnormal DV flow at the time of first trimester screening is associated to an increased risk of CHD both in unselected and in euploid fetuses, with a RR of 6.4. The prevalence of DV abnormalities in fetuses with CHD varied widely, depending on the population screened. In our meta-analysis, the LR + of CHD in case of DV abnormalities was 6.4 in euploid fetus while was 1.8 in the high-risk population. The DV examination demonstrated a good diagnostic accuracy for CHD detection in euploid fetuses, but it was poor in the high-risk and in the unselected population.

### Interpretation of the Findings

In a previous meta-analysis published in 2011 [20], authors found that DV waveform in fetuses with normal NT has a low sensitivity (19%) for the detection of CHD, and around 96% of fetuses with normal NT and normal heart have normal DV. In this previous review, only 9 papers were included in the final analysis. The results from the present meta-analyses support and confirm the findings of the previous one, relying on the evidence from 22 studies available in the current literature.

According to our findings, the DV for the detection of CHD performed better in the sub-population of euploid fetuses, with a sensitivity of 50% [95% CI 0.35–0.65] and a specificity of 92% [95% CI 0.86–0.96]. This is an interesting finding, as the euploid population is typically considered to be less likely to be affected by CHD. However, an abnormal DV finding in this low-risk population, would indicate the need for a detailed cardiac assessment. This strategy could hopefully increase the prenatal detection of CHD.

It has been extensively reported that many indirect signs such as increased NT, TR, and abnormal DV are associated with a significantly increased risk of CHD [55, 56]. The underlying mechanism of this association is still unclear. However, the presence of these markers might represent a certain degree of cardiac impairment, which becomes evident only in the first trimester of pregnancy, when the placental resistances are higher and fetal heart compliance is reduced [57]. Another previous meta-analysis [23] on the role of fetal tricuspid regurgitation (TR) in the first trimester as a screening marker for CHD, demonstrated that the association between TR and CHD is higher for cases with increased risk for cardiac defects, as in case of increased NT, while there is no association in the low-risk population. TR shows good performances in screening for CHDs in high-risk fetuses, but lacks clear evidence thereof in low-risk populations. On the contrary, DV seems to perform better in fetuses with a low risk of CHD. These findings may suggest that the best way to select women at higher risk of CHD is a complementary assessment of both the DV and the TR in the first trimester.

Several studies published to date focused on the detection of CHD at the time of the first trimester screening based on ultrasound markers. The improvement in the available technology with the use of high-resolution transducers and color-flow mapping, has allowed the evaluation of the fetal heart at the time of the NT scan, despite the small size of anatomical structures at this stage of pregnancy [58, 59]. A combined evaluation of the 4-chamber and 3-vessel and trachea views in color mapping was reported to have a sensitivity of 88.57% and specificity of 100% for detection of CHD in an unselected population [59]. An earlier ultrasound cardiac assessment performed in a selected population by experienced operators is associated with several advantages: early detection and exclusion of major CHD, early reassurance to at-risk mothers, earlier genetic diagnosis and counselling, and easier pregnancy termination if requested. However, a first trimester cardiac assessment is also associated with some limitations, including: the need for trained and experienced operators performing the exam, uncertain cost/benefit ratio, difficult counselling due to the unclear significance of the ultrasound findings, and late development of some anatomical structures and malformations (e.g. coarctation of the aorta, hypoplastic left heart), which make early detection impossible [60].

Currently, there are no international recommendations to perform a fetal cardiac evaluation when an abnormal DV is detected in the first trimester. In such scenario, the decision to perform an additional cardiac evaluation, especially in those with a normal karyotype, should be tailored on the individualized risk assessment considering all the available risk factors. The Fetal Medicine Foundation recommends a detailed ultrasound examination to diagnose or exclude major CHD if abnormal DV or TR are detected.

Cell-free DNA testing (also called non-invasive prenatal testing—NIPT) is currently available in many countries. The widespread diffusion of the NIPT has led to a reduction of the role of the first trimester ultrasound, as the residual risk of chromosomal abnormality is very low in women who choose to have NIPT as their primary screening test in the first trimester of pregnancy. Despite this, many international societies have underlined the importance of the first trimester ultrasound, despite a negative NIPT screening. Indeed, enlarged fetal NT is also associated with genetic syndromes and fetal structural abnormalities such as cardiac, abdominal wall, and musculoskeletal malformations in addition to fetal chromosomal abnormality. In addition, the finding of a particularly increased NT thickness represents an indication for fetal karyotyping and micro-array analyses. Women choosing to have NIPT as a primary screening test should still be offered the opportunity to undergo an 11–13 week ultrasound for an early structural assessment, as around 45% of major abnormalities can now be detected at this gestation, with even higher detection rates for the lethal abnormalities [61]. Our findings are in line with this consideration. The finding of an abnormal DV in the first trimester should raise the suspicion of CHD and should prompt a detailed ultrasound assessment of “early” fetal anatomy.

### Strength and limitations

This systematic review represents an update of a previous one published in 2011. In the previous review nine studies assessing the performance of ductus venosus for the detection of CHD were included in the final analysis. However, in the last decade several articles have been published on this topic, allowing us to identify 13 more studies eligible for the present study, allowing us to rely on 22 studies for our analysis. Inclusion of a higher number of studies is one of the strengths of our systematic review. Furthermore, our study design relied on a comprehensive search strategy providing quantitative pooling of the data using meta-analysis of proportion and HSROC. All the included studies had an overall reasonably good methodology, as showed by the quality assessment tools. In addition to this, the DV examination was performed by sonographers or physicians according to the recommendations of The Fetal Medicine Foundation, therefore, fulfilling strict criteria which allowed us to rely on accurate data leading to our results.

One of the limitations of the present study is the high heterogeneity found between the study included, most likely due to the differences between the populations involved in each study (unselected, euploid, and high-risk fetuses) and to the different definitions of abnormal DV. Such issue might limit the robustness of the reported evidence and promote the need for additional, well-designed, prospective studies.

## Conclusion

Abnormal DV in the first trimester increases the risk of CHD with a moderate sensitivity for euploid fetuses. Therefore, the combination of DV examination with other markers (NT, TV regurgitation) in the first trimester, could be helpful to identify fetuses otherwise considered to be at low risk for CHD. This strategy, in addition to the improvement of the fetal heart examination in the first trimester, can increase the detection of major CHD at earlier stage of pregnancy.

## Supplementary Information

Below is the link to the electronic supplementary material.

Supplementary file1 Deeks’ Funnel Plot Asymmetry Test for overall fetuses (JPG 151 KB)

Supplementary file2 Deeks’ Funnel Plot Asymmetry Test for overall fetuses for unselected fetuses (JPG 130 KB)

Supplementary file3 Deeks’ Funnel Plot Asymmetry Test for overall fetuses for euploid fetuses (JPG 78 KB)

Supplementary file4 Deeks’ Funnel Plot Asymmetry Test for overall fetuses for high-risk fetuses (JPG 116 KB)

Supplementary file5 Deeks’ Funnel Plot Asymmetry Test for overall fetuses for normal NT fetuses (JPG 77 KB)

Supplementary file6 Excluded studies with reason for exclusion (DOCX 125 KB)

Supplementary file7 Summary of Risk of Bias and Applicability Concerns according to QUADAS-2 score (DOCX 73 KB)

## Data Availability

The data that support this systematic review and meta-analysis are available from the corresponding author, upon reasonable request.
