# Supplementary figures and images for: The diagnostic performance of the ductus venosus for the detection of cardiac defects in the first trimester: a systematic review and diagnostic test accuracy meta-analysis
Source: Arch Gynecol Obstet. 2022 Oct 31;308(2):435–51. doi: 10.1007/s00404-022-06812-w (PMC10293352; doi:10.1007/s00404-022-06812-w)

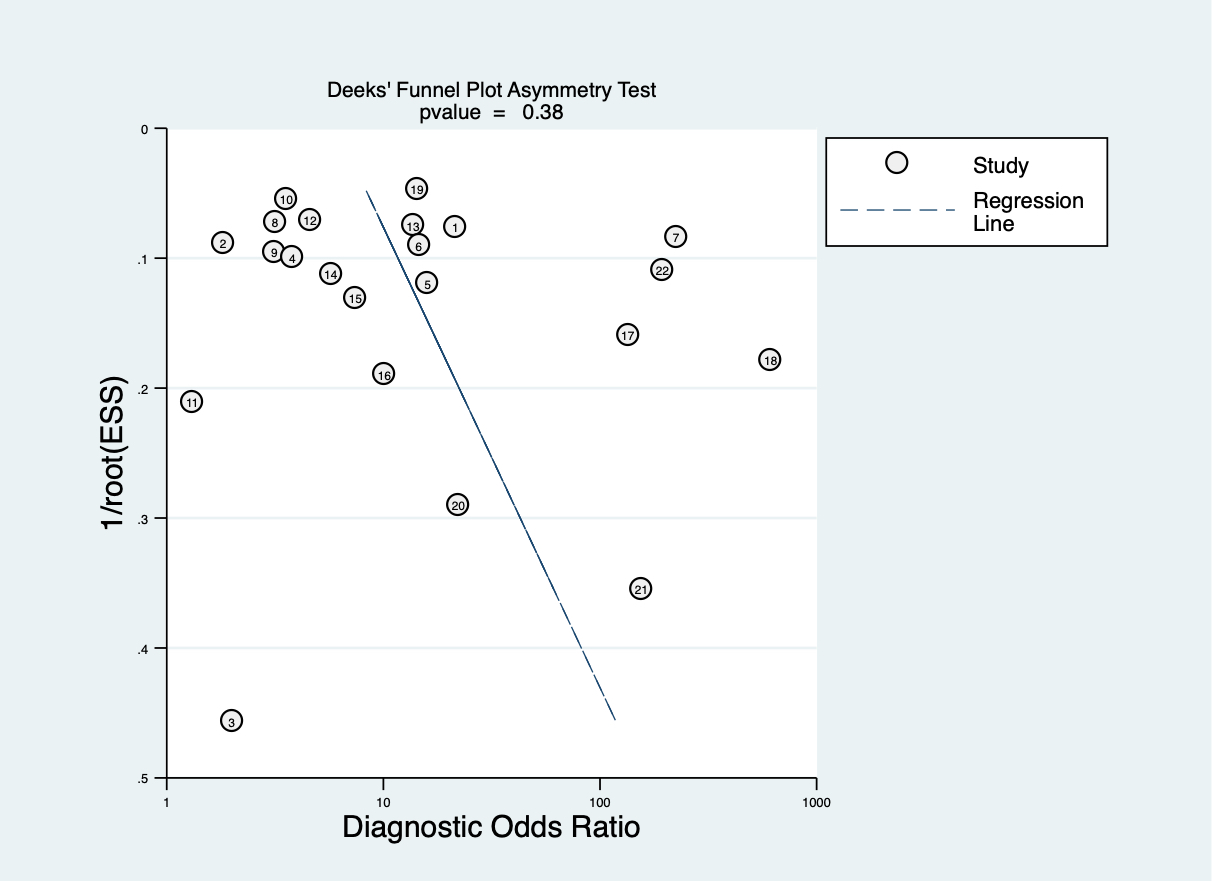

Supplement: Supplementary file 1 — Supplementary file1 Deeks’ Funnel Plot Asymmetry Test for overall fetuses (JPG 151 KB) [file 404_2022_6812_MOESM1_ESM.jpg]

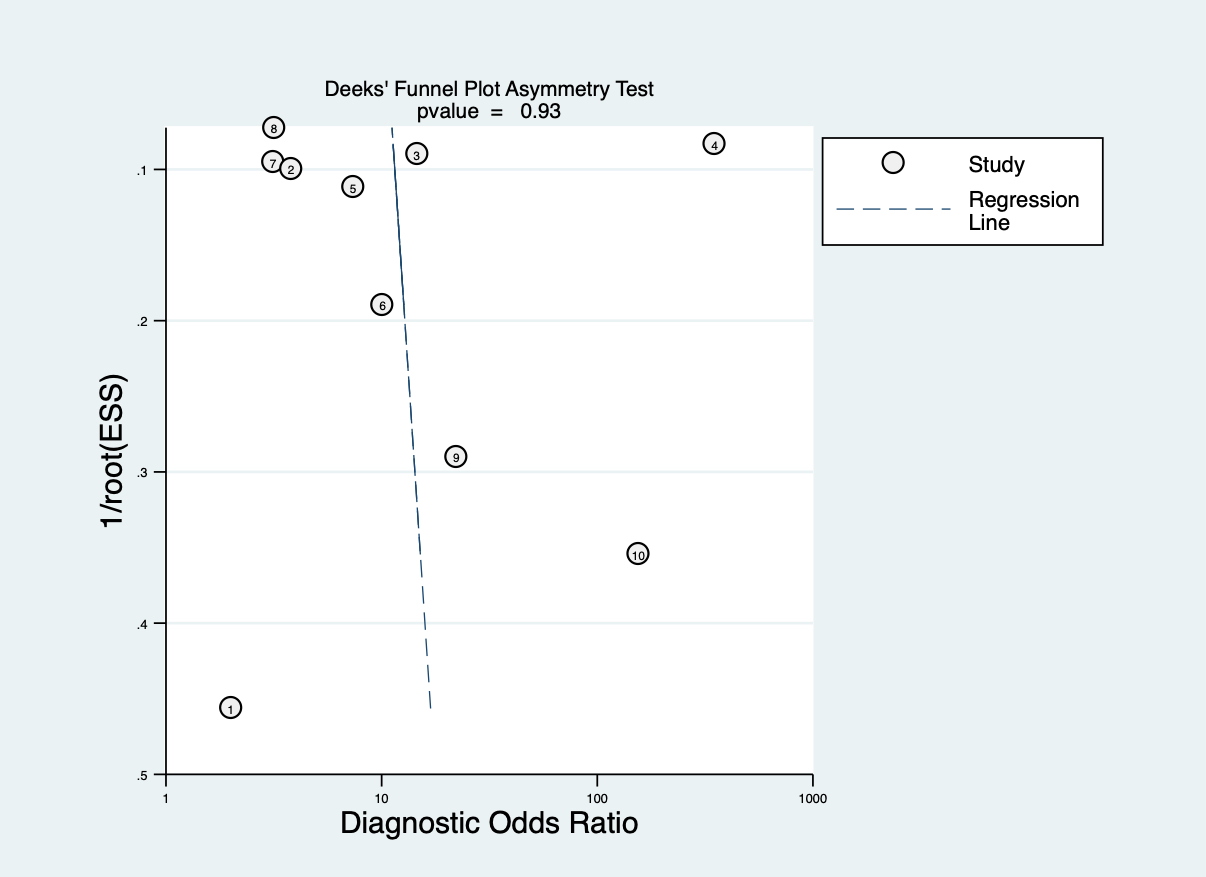

Supplement: Supplementary file 2 — Supplementary file2 Deeks’ Funnel Plot Asymmetry Test for overall fetuses for unselected fetuses (JPG 130 KB) [file 404_2022_6812_MOESM2_ESM.jpg]

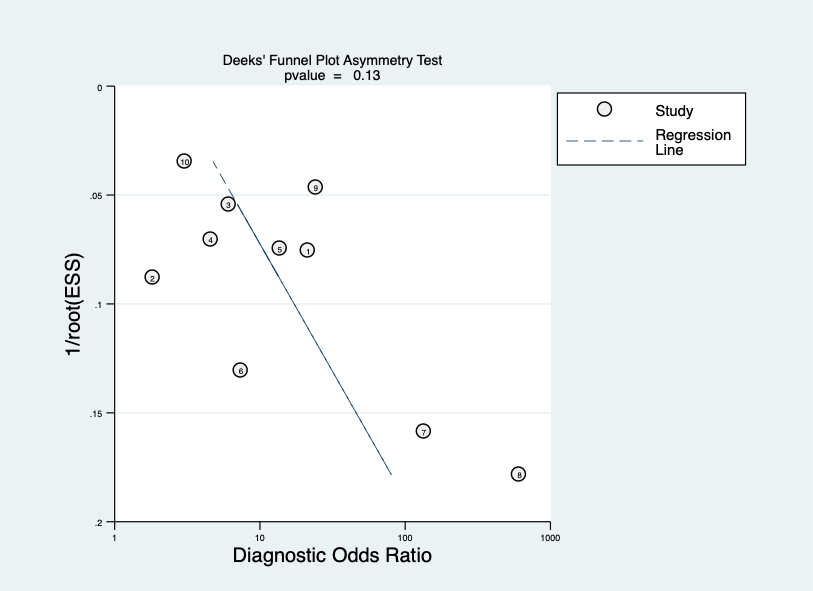

Supplement: Supplementary file 3 — Supplementary file3 Deeks’ Funnel Plot Asymmetry Test for overall fetuses for euploid fetuses (JPG 78 KB) [file 404_2022_6812_MOESM3_ESM.jpg]

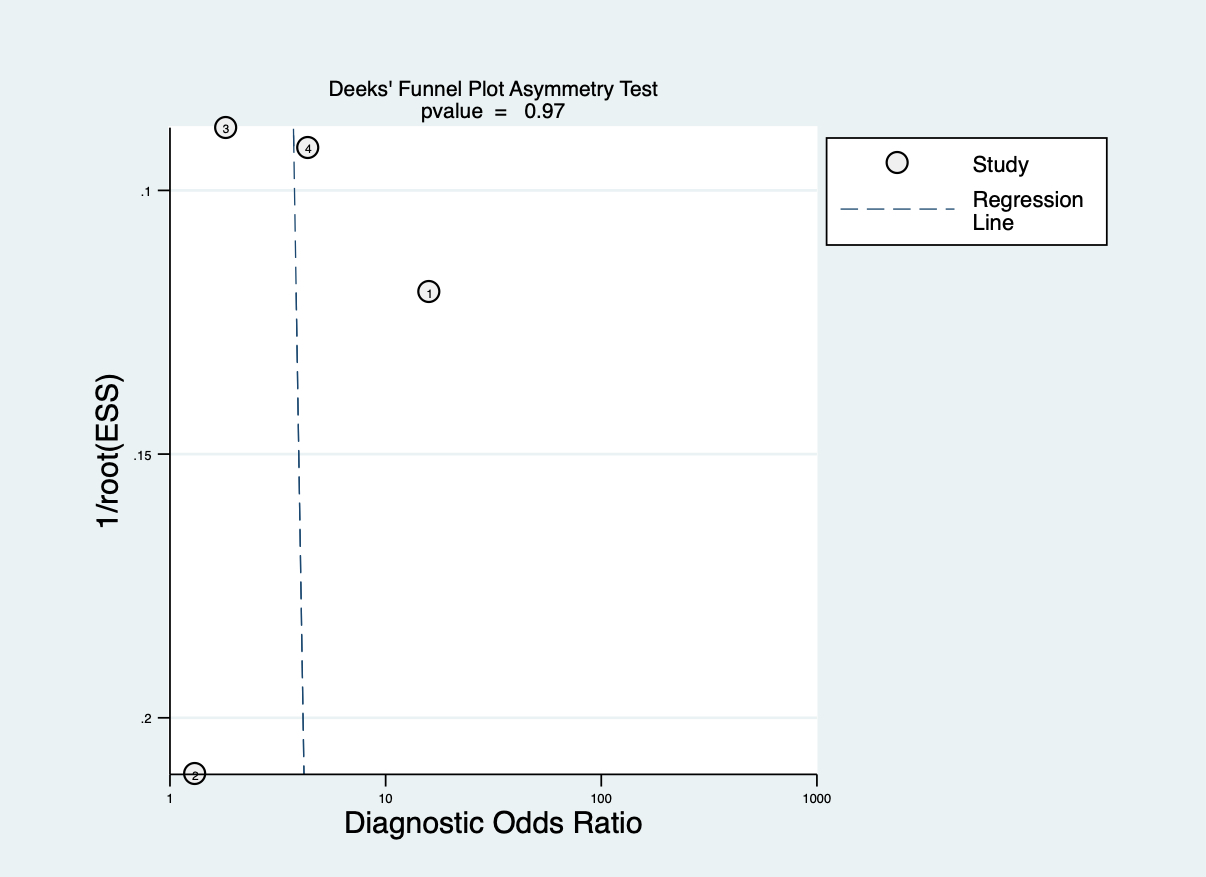

Supplement: Supplementary file 4 — Supplementary file4 Deeks’ Funnel Plot Asymmetry Test for overall fetuses for high-risk fetuses (JPG 116 KB) [file 404_2022_6812_MOESM4_ESM.jpg]

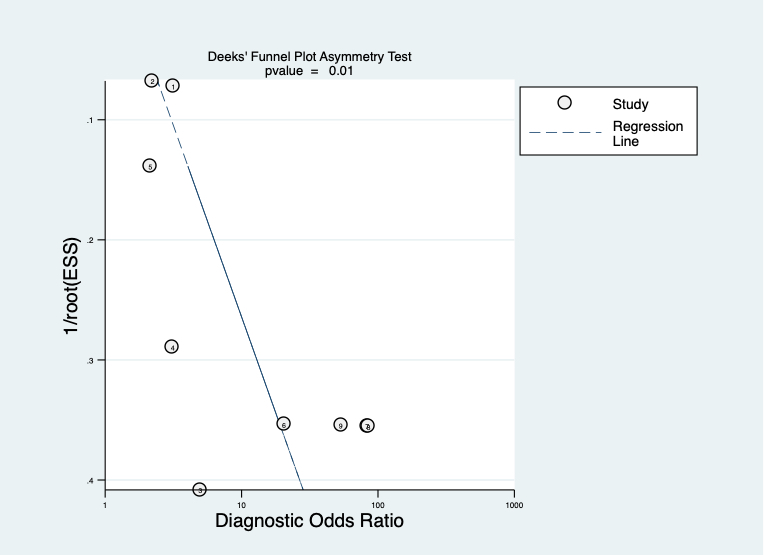

Supplement: Supplementary file 5 — Supplementary file5 Deeks’ Funnel Plot Asymmetry Test for overall fetuses for normal NT fetuses (JPG 77 KB) [file 404_2022_6812_MOESM5_ESM.jpg]
