# Supplementary material for: The diagnostic performance of the ductus venosus for the detection of cardiac defects in the first trimester: a systematic review and diagnostic test accuracy meta-analysis
Source: Arch Gynecol Obstet. 2022 Oct 31;308(2):435–51. doi: 10.1007/s00404-022-06812-w (PMC10293352; doi:10.1007/s00404-022-06812-w)
Supplement: Supplementary file 6 — Supplementary file6 Excluded studies with reason for exclusion (DOCX 125 KB) [file 404_2022_6812_MOESM6_ESM.docx]

| **AUTHOR** | **STUDY** | **JOURNAL** | **EXCLUDED** |
| --- | --- | --- | --- |
| Ferrazzi E, Lees C, Acharya G | The controversial role of the ductus venosus in hypoxic human fetuses | Acta Obstet Gynecol Scand. 2019 | NOT FIRST TRIMESTER OF PREGNANCY |
| Erenel H, Karsli MF, Ozel A, Korkmaz SO, Sen C. | Ductus venosus-systemic shunt. Report of six cases and systematic review of the literature. | J Matern Fetal Neonatal Med. 2019 | REVIEW/ OPINION ARTICLE |
| Braga M, Moleiro ML, Guedes-Martins L. | Clinical Significance of Ductus Venosus Waveform as Generated by Pressure- volume Changes in the Fetal Heart | Curr Cardiol Rev. 2019 | REVIEW/ OPINION ARTICLE |
| Wu H, Tao G, Cong X, Li Q, Zhang J, Ma Z, Zhang Z | Prenatal sonographic characteristics and postnatal outcomes of umbilical-portal-systemic venous shunts under the new in-utero classification: A retrospective study | Medicine (Baltimore). 2019 | NO RELEVANT TO STUDY QUESTION |
| Tang Y, Luo H, Mu D, Yang T, Zhu Q, Yang F, Liu G | Early diagnosis of trisomy 21, trisomy 18 and trisomy 13 using nuchal translucency thickness and ductus venosus blood flow waveform in West China | Mol Med Rep. 2019 | NO RELEVANT TO STUDY QUESTION |
| Poeppelman RS, Tobias JD | Patent Ductus Venosus and Congenital Heart Disease: A Case Report and Review. | Cardiol Res. 2018 | REVIEW/ OPINION ARTICLE |
| Tekesin I. | The Value of Detailed First-Trimester Ultrasound Anomaly Scan for the Detection of Chromosomal Abnormalities. | Ultraschall Med. 2018 | NO RELEVANT TO STUDY QUESTION |
| Kalayci H, Yilmaz Baran Ş, Doğan Durdağ G, Yetkinel S, Alemdaroğlu S, Özdoğan S, Yüksel Şimşek S, Bulgan Kiliçdağ E | Reference values of the ductus venosus pulsatility index for pregnant women between 11 and 13+6 weeks of gestation | J Matern Fetal Neonatal Med. 2018 | NO RELEVANT TO STUDY QUESTION |
| Nemescu D, Bratie A, Mihaila A, Navolan D, Tanase A. | [First trimester combined screening for fetal aneuploidies enhanced with additional ultrasound markers: an 8-year prospective study.](https://www.ncbi.nlm.nih.gov/pubmed/29781076) | Ginekol Pol. 2018 | NO RELEVANT TO STUDY QUESTION |
| Turan S, Turan OM | Harmony Behind the Trumped-Shaped Vessel: the Essential Role of the Ductus Venosus in Fetal Medicine. | Balkan Med J. 2018 | REVIEW/ OPINION ARTICLE |
| Wagner P, Sonek J, Eberle K, Abele H, Hoopmann M, Prodan N, Kagan KO | First trimester screening for major cardiac defects based on the ductus venosus flow in fetuses with trisomy 21 | Prenat Diagn. 2018 | THE SAME POPULATION AS ANOTHER STUDY |
| Philipp Wagner*, Jiri Sonek**, Jessika Klein*, Markus Hoopmann*, Harald Abele* and Karl Oliver Kagan* | First trimester ultrasound screening for trisomy 21 based on maternal age, fetal nuchal translucency and different methods of ductus venosus assessment | Prenat Diagn. 2017 | NO RELEVANT TO STUDY QUESTION |
| Czuba B, Zarotyński D, Dubiel M, Borowski D, Węgrzyn P, Cnota W, Reska-Nycz M, Mączka M, Wielgoś M, Sodowski K, Serafin D, Kubaty A, Bręborowicz GH | Screening for trisomy 21 based on maternal age, nuchal translucency measurement, first trimester biochemistry and quantitative and qualitative assessment of the flow in the DV - the assessment of efficacy | Ginekol Pol. 2017 | NO RELEVANT TO STUDY QUESTION |
| Hidaka N, Sato Y, Kido S, Fujita Y, Kato K | Ductus venosus Doppler and the postnatal outcomes of growth restricted fetuses with absent end-diastolic blood flow in the umbilical arteries | Taiwan J Obstet Gynecol. 2017 | NOT FIRST TRIMESTER OF PREGNANCY |
| Karakoç G, Yavuz A, Eriş Yalçın S, Akkurt MÖ, Danışman N | [The significance of reverse flow in ductus venosus between sixteen and twenty weeks' gestation.](https://www.ncbi.nlm.nih.gov/pubmed/28913131) | Turk J Obstet Gynecol. 2017 | NO RELEVANT TO STUDY QUESTION |
| Hernandez-Andrade E, Patwardhan M, Cruz-Lemini M, Luewan S. | Early Evaluation of the Fetal Heart. | Fetal Diagn Ther. 2017 | REVIEW/ OPINION ARTICLE |
| Alldred SK, Takwoingi Y, Guo B, Pennant M, Deeks JJ, Neilson JP, Alfirevic Z | First trimester ultrasound tests alone or in combination with first trimester serum tests for Down's syndrome screening | Cochrane Database Syst Rev. 2017 | REVIEW/ OPINION ARTICLE |
| Kaul A, Singh C, Gupta R, Arora N, Gupta A. | Observational study comparing the performance of first-trimester screening protocols for detecting trisomy 21 in a North Indian population. | Int J Gynaecol Obstet. 2017 | NO RELEVANT TO STUDY QUESTION |
| Korkalainen N, Räsänen J, Kaukola T, Kallankari H, Hallman M, Mäkikallio K. | Fetal hemodynamics and adverse outcome in primary school-aged children with fetal growth restriction: a prospective longitudinal study | Acta Obstet Gynecol Scand. 2017 | NOT FIRST TRIMESTER OF PREGNANCY |
| Wiechec M, Knafel A, Nocun A, Wiercinska E, Ludwin A, Ludwin I. | What are the most common first-trimester ultrasound findings in cases of Turner syndrome? | J Matern Fetal Neonatal Med. 2017 | NO RELEVANT TO STUDY QUESTION |
| Fouron JC, McNeal-Davidson A, Abadir S, Fournier A, Bigras JL, Boutin C, Brassard M, Raboisson MJ, van Doesburg N, Berger A, Brisebois S, Gendron R. | Prenatal diagnosis and prognosis of accelerated idioventricular rhythm. | Ultrasound Obstet Gynecol. 2017 | NOT FIRST TRIMESTER OF PREGNANCY |
| Gottschalk I, Jehle C, Herberg U, Breuer J, Brockmeier K, Bennink G, Hellmund A, Strizek B, Gembruch U, Geipel A, Berg C. | Prenatal diagnosis of absent pulmonary valve syndrome from first trimester onwards: novel insights into pathophysiology, associated conditions and outcome. | Ultrasound Obstet Gynecol. 2017 | NO RELEVANT TO STUDY QUESTION |
| Burger NB, Haak MC, Kok E, de Groot CJ, Shou W, Scambler PJ, Lee Y, Cho E, Christoffels VM, Bekker MN | Cardiac defects, nuchal edema and abnormal lymphatic development are not associated with morphological changes in the ductus venosus | Early Hum Dev. 2016 | ANIMAL STUDIES |
| Wald NJ, Bestwick JP | [Prenatal reflex DNA screening for Down syndrome: enhancing the screening performance of the initial first trimester test.](https://www.ncbi.nlm.nih.gov/pubmed/26841247) | Prenat Diagn. 2016 | NO RELEVANT TO STUDY QUESTION |
| Wagner P, Sonek J, Hoopmann M, Abele H, Kagan KO. | First-trimester screening for trisomies 18 and 13, triploidy and Turner syndrome by detailed early anomaly scan | Ultrasound Obstet Gynecol. 2016 | DATA EXTRACTION NOT POSSIBLE |
| Dahlbäck C, Gudmundsson S. | [Increased pulsatility in the fetal ductus venosus is not related to altered cardiac strain in high-risk pregnancies.](https://www.ncbi.nlm.nih.gov/pubmed/26371879) | J Matern Fetal Neonatal Med. 2016 | NOT FIRST TRIMESTER OF PREGNANCY |
| Chiu WH, Lee SM, Tung TH, Tang XM, Liu RS, Chen RC | Length to width ratio of the ductus venosus in simple screening for fetal congenital heart diseases in the second trimester | Medicine (Baltimore). 2016 | NOT FIRST TRIMESTER OF PREGNANCY |
| Rezaee T, Hassani K | Numerical investigation of the haemodynamics in the human fetal umbilical vein/ductus venosus based on the experimental data. | Biosci Rep. 2016 | NO RELEVANT TO STUDY QUESTION |
| Matias A, Montenegro N. | Ductus venosus in twins: science matters. | Ultrasound Obstet Gynecol. 2016 | NO RELEVANT TO STUDY QUESTION |
| İlhan G, İyibozkurt AC, Kalelioğlu Hİ, İbrahimoğlu L, Zebitay AG, Eken MK, Karasu AF. | Effects of fetal cardiac anomalies on ductus venosus and aortic isthmus doppler profiles. | Arch Gynecol Obstet. 2016 | NOT FIRST TRIMESTER OF PREGNANCY |
| Seravalli V, Miller JL, Block-Abraham D, Baschat AA. | Ductus venosus Doppler in the assessment of fetal cardiovascular health: an updated practical approach. | Acta Obstet Gynecol Scand. 2016 | REVIEW/ OPINION ARTICLE |
| Yu R, Li SL, Luo GY, Wen HX, Ouyang SY, Chen CY, Yuan Y | First-Trimester Echocardiographic Features and Perinatal Outcomes in Fetuses With Congenital Absence of the Aortic Valve. | J Ultrasound Med. 2016 | NO RELEVANT TO STUDY QUESTION |
| Avcı ME, Yozgat Y, Şanlıkan F, Yıldırım G, Polat İ, Karaarslan U. | Utility of ductus venosus blood flow in the study of cardiac function in fetuses with intracardiac echogenic focus. | J Clin Ultrasound. 2016 | NOT FIRST TRIMESTER OF PREGNANCY |
| Qian Y, Bing H, Ailu C, Wei S, Yu W, Lei W. | Abnormal Connection of the Ductus Venosus to a Dilated Coronary Sinus Imaged by Prenatal Echocardiography: Case Report. | Echocardiography. 2016 | NOT FIRST TRIMESTER OF PREGNANCY |
| İlhan, Gülşah 1 ; İyibozkurt, Ahmet Cem 2 ; Kalelioğlu, Halil İbrahim 2 ; İbrahimoğlu, Lemi 2 ; Zebitay, Ali Galip 1 ; Eken, Meryem Kürek 3 ; Karasu, Ayşe Filiz Gökmen | Effects of fetal cardiac anomalies on ductus venosus and aortic isthmus doppler profiles | Archives of gynecology and obstetrics.2016 | NOT FIRST TRIMESTER OF PREGNANCY |
| Abele, Harald 1 ; Wagner, Philipp 1 ; Sonek, Jiri 2 ; Hoopmann, Markus 1 ; Brucker, Sara 1 ; Artunc-Ulkumen, Burcu 3 ; Kagan, Karl Oliver 1 | First trimester ultrasound screening for Down syndrome based on maternal age, fetal nuchal translucency and different combinations of the additional markers nasal bone, tricuspid and ductus venosus flow | Prenatal diagnosis 35.12 (Dec 2015): 1182-6 | NO RELEVANT TO STUDY QUESTION |
| Seckin, K.D. 1 ; Karslı, M.F. 1 ; Baser, E. 1 ; Yeral, M.I. 1 ; Tasin, C. 1 ; Ozgu Erdinc, A.S. 1 ; Danisman | Obstetric outcomes in pregnancies with normal nuchal translucency and abnormal ductus venosus Doppler in the first trimester ultrasonography | Journal of Obstetrics and Gynaecology | NO RELEVANT TO STUDY QUESTION |
| Togrul, Cihan 1 ; Ozaksit, Gulnur M. 1 ; Seckin, Kerem Doga 1 ; Baser, Eralp 1 ; Karsli, Mehmet F. 1 ; Gungor, Tayfun | Is there a role for fetal ductus venosus and hepatic artery Doppler in screening for fetal aneuploidy in the first trimester? | Journal of Maternal-Fetal and Neonatal Medicine | NO RELEVANT TO STUDY QUESTION |
| Neocleous, Andreas; Nicolaides, Kypros; Schizas, Christos | First Trimester Non-invasive Prenatal Diagnosis: A Computational Intelligence Approach | IEEE journal of biomedical and health informatics | NO RELEVANT TO STUDY QUESTION |
| İlhan, Gülşah 1 ; İyibozkurt, Ahmet Cem 2 ; Kalelioğlu, Halil İbrahim 2 ; İbrahimoğlu, Lemi 2 ; Zebitay, Ali Galip 1 ; Eken, Meryem Kürek 3 ; Karasu, Ayşe Filiz Gökmen | Effects of fetal cardiac anomalies on ductus venosus and aortic isthmus doppler profiles | Archives of Gynecology and Obstetrics | NOT FIRST TRIMESTER OF PREGNANCY |
| Oviedo-Cruz, Héctor; Hernández-Paredez, Javier; Ruíz-Ramírez, Areysha Vanessa | Prenatal screening for anueploidies in the first trimester: Audit to a Fetal Medicine Centre with specialized Laboratory in Mexico | Ginecología y obstetricia de México | NO RELEVANT TO STUDY QUESTION |
| Schenone, Aldo L 1 ; Giugni, G 2 ; Schenone, M H 3 ; Diaz, L 2 ; Bermudez, A 2 ; Majdalany, D 4 ; Sosa-Olavarria | Case Series: Fetal Pulmonary Vein A-Wave Reversal: An Early Marker of Left-Sided Cardiac Anomalies? | AJP reports | NO RELEVANT TO STUDY QUESTION |
| Sarkola, Taisto 1 ; Ojala, Tiina H. 1 ; Ulander, Veli-Matti 2 ; Jaeggi, Edgar 3 ; Pitkänen, Olli M. | Screening for congenital heart defects by transabdominal ultrasound - Role of early gestational screening and importance of operator training | Acta Obstetricia et Gynecologica Scandinavica | REVIEW/ OPINION ARTICLE |
| Clur, Sally-Ann B. 1 ; Bilardo, Caterina M. | Early detection of fetal cardiac abnormalities: how effective is it and how should we manage these patients? | Prenatal diagnosis | REVIEW/ OPINION ARTICLE |
| Nicolaides, K.H. 1 ; Syngelaki, A. 2 ; Poon, L.C. 2 ; Gil, M.M. 2 ; Wright, | First-trimester contingent screening for trisomies 21, 18 and 13 by biomarkers and maternal blood cell-free DNA testing | Fetal Diagnosis and Therapy | NO RELEVANT TO STUDY QUESTION |
| Arya, Bhawna 1 ; Krishnan, Anita 1 ; Donofrio, Mary T. | Clinical utility of ductus venosus flow in fetuses with right-sided congenital heart disease | Journal of ultrasound in medicine | NOT FIRST TRIMESTER OF PREGNANCY |
| Cuckle, Howard | Prenatal screening using maternal markers | Journal of Clinical Medicine | REVIEW/ OPINION ARTICLE |
| Tsyv'ian, P B; Kovalev, V V; Kosovtsova, N V | Ultrasound markers of the genetic pathology and early hemodynamic changes in human embryo | Fiziologiia cheloveka | DATA EXTRACTION NOT POSSIBLE |
| Herrera, Tania T 1 ; Sinisterra, Scarlett 2 ; Solis, Alcibiades 1 ; Britton, Gabrielle B 3 | First trimester screening using ultrasound and serum markers in Panamanians: Factors associated with adverse pregnancy outcomes | Journal of research in medical sciences | DATA EXTRACTION NOT POSSIBLE |
| Sanapo, L. 1 ; Turan, O.M. 1 ; Turan, S. 1 ; Ton, J. 1 ; Atlas, M. 1 ; Baschat | Correlation analysis of ductus venosus velocity indices and fetal cardiac function | Ultrasound in obstetrics & gynecology | NOT FIRST TRIMESTER OF PREGNANCY |
| Gembruch, U. 1 ; Kempe, A. 1 ; Hellmund, A. 1 ; Rösing, B. 1 ; Willruth, A. 1 ; Berg, C. 1 ; Geipel | The diagnostics of fetal heart defects in the first and early second trimester - Early fetal echocardiography | Geburtshilfe und Frauenheilkunde | REVIEW/ OPINION ARTICLE |
| Khalil, Asma 1 ; Nicolaides, Kypros H | Fetal heart defects: potential and pitfalls of first-trimester detection | Seminars in fetal & neonatal medicine | REVIEW/ OPINION ARTICLE |
| Dahlbäck, C. 1 ; Pihlsgård, M. 1 ; Gudmundsson, S. | Abnormal ductus venosus pulsatility index in the absence of concurrent umbilical vein pulsations does not indicate worsening fetal condition | Ultrasound in obstetrics & gynecology | NOT FIRST TRIMESTER OF PREGNANCY |
| Rembouskos, G 1 ; Passamonti, U; De Robertis, V; Tempesta, A; Campobasso, G; Volpe, G; Gentile, M; Volpe | Aberrant right subclavian artery (ARSA) in unselected population at first and second trimester ultrasonography | Prenatal diagnosis | NO RELEVANT TO STUDY QUESTION |
| Geipel, A 1 ; Gembruch, | Screening performance of first trimester nuchal translucency, ductus venosus blood flow and tricuspid regurgitation for cardiac defects | Zeitschrift für Geburtshilfe und Neonatologie | REVIEW/ OPINION ARTICLE |
| Demirturk, Fazli 1 ; Caliskan, Ahmet Cantug 2 ; Aytan, Hakan 3 ; Sahin, Semsettin | A preliminary retrospective study about the relationship between ductus venosus Doppler indices, nuchal translucency (NT) and biochemical markers in the first and second trimester screening tests | Gynecological Endocrinology | NO RELEVANT TO STUDY QUESTION |
| Baschat, A A; Turan, O M; Turan, S | Ductus venosus blood-flow patterns: more than meets the eye? | Ultrasound in obstetrics & gynecology | REVIEW/ OPINION ARTICLE |
| Pereira, Susana 1 ; Ganapathy, Ramesh 1 ; Syngelaki, Argyro 1 ; Maiz, Nerea 1 ; Nicolaides, Kypros H | Contribution of fetal tricuspid regurgitation in first-trimester screening for major cardiac defects | Obstetrics and Gynecology | THE SAME POPULATION AS ANOTHER STUDY |
| Clur, S A B 1 ; Oude Rengerink, K; Mol, B W J; Ottenkamp, J; Bilardo, | Fetal cardiac function between 11 and 35 weeks' gestation and nuchal translucency thickness | Ultrasound in obstetrics & gynecology | NO RELEVANT TO STUDY QUESTION |
| Timmerman, E 1 ; Oude Rengerink, K; Pajkrt, E; Opmeer, B C; van der Post, J A M; Bilardo, | Ductus venosus pulsatility index measurement reduces the false-positive rate in first-trimester screening | Ultrasound in obstetrics & gynecology | NO RELEVANT TO STUDY QUESTION |
| de Mooij, Yolanda M 1 ; Haak, Monique C; Bartelings, Margot M; Twisk, Jos W; Gittenberger-de Groot, Adriana; van Vugt, John M G; Bekker, Mireille N | Abnormal ductus venosus flow in first-trimester fetuses with increased nuchal translucency: relationship with the type of cardiac defect? | Journal of ultrasound in medicine | DATA EXTRACTION NOT POSSIBLE |
| Ozkaya, Okan 1 ; Sezik, Mekin; Ozbasar, Demir; Kaya, Hakan | Abnormal ductus venosus flow and tricuspid regurgitation at 11-14 weeks' gestation have high positive predictive values for increased risk in first-trimester combined screening test: results of a pilot study | Taiwanese journal of obstetrics & gynecology | NO RELEVANT TO STUDY QUESTION |
| Zhang, Ming 1 ; Pu, Da-Rong 1 ; Zhou, Qi-Chang 1 ; Peng, Qing-Hai 1 ; Tian, Lei-Qi | Four-dimensional echocardiography with B-flow imaging and spatiotemporal image correlation in the assessment of congenital heart defects | Prenatal Diagnosis | NOT FIRST TRIMESTER OF PREGNANCY |
| Baś-Budecka, Elzbieta 1 ; Perenc, Małgorzata; Sieroszewski, Piotr 1 Klinika | The role of fetal nuchal translucency (NT) and ductus venosus blood flow (DV) in the detection of congenital heart defects | Ginekologia polska | DATA EXTRACTION NOT POSSIBLE |
| Maiz, Nerea 1 ; Nicolaides, Kypros H 1 Fetal Medicine Unit, Centro Sanitario Virgen del Pilar, P. Zarategui | Ductus venosus in the first trimester: contribution to screening of chromosomal, cardiac defects and monochorionic twin complications | Fetal diagnosis and therapy | REVIEW/ OPINION ARTICLE |
| Własienko, Paweł 1 ; Hamela-Olkowska, Anita; Jalinik, Katarzyna; Dangel, Joanna | The possibility of cardiovascular system evaluation in fetuses at 11.0 to 13.6 weeks of gestation in a reference perinatal cardiology centre |  | DATA EXTRACTION NOT POSSIBLE |
| Gollo, Carlos Alberto 1 ; Murta, Carlos Geraldo Viana 2 ; Bussamra, Luiz Cláudio 3 ; Santana, Renato Martins 3 ; Moron, Antônio Fernandes | Predictive value for fetal outcome of Doppler velocimetry of the ductus venosus between the 11^th^ and the 14^th^ gestation week | Revista Brasileira de Ginecologia e Obstetricia | DATA EXTRACTION NOT POSSIBLE |
| Oh, C. 1 ; Harman, C. 1 ; Baschat | Abnormal first-trimester ductus venosus blood flow: A risk factor for adverse outcome in fetuses with normal nuchal translucency | Ultrasound in Obstetrics and Gynecology | NO RELEVANT TO STUDY QUESTION |
| Berg, C. 1 ; Kremer, C. 2 ; Geipel, A. 2 ; Kohl, T. 2 ; Germer, U. 3 ; Gembruch, | Ductus venosus blood flow alterations in fetuses with obstructive lesions of the right heart | Ultrasound in Obstetrics and Gynecology | NOT FIRST TRIMESTER OF PREGNANCY |
| Haak, Monique C 1 ; Twisk, Jos W W R; Bartelings, Margot M; Gittenberger-de Groot, Adriana C; van Vugt, John M | Ductus venosus flow velocities in relation to the cardiac defects in first-trimester fetuses with enlarged nuchal translucency | American journal of obstetrics and gynecology | DATA EXTRACTION NOT POSSIBLE |
| Bilardo, C M 1 ; Müller, M A; Zikulnig, L; Schipper, M; Hecher | Ductus venosus studies in fetuses at high risk for chromosomal or heart abnormalities: relationship with nuchal translucency measurement and fetal outcome | Ultrasound in obstetrics & gynecology | DATA EXTRACTION NOT POSSIBLE |
| Matias, A 1 ; Huggon, I; Areias, J C; Montenegro, N; Nicolaides, K H 1 Harris | Cardiac defects in chromosomally normal fetuses with abnormal ductus venosus blood flow at 10-14 weeks | Ultrasound in obstetrics & gynecology | THE SAME POPULATION AS ANOTHER STUDY |
| Carvalho | Nuchal translucency, ductus venosus and congenital heart disease: an important association--a cautious analysis | Ultrasound in obstetrics & gynecology | REVIEW/ OPINION ARTICLE |
