# Supplementary material for: The diagnostic performance of the ductus venosus for the detection of cardiac defects in the first trimester: a systematic review and diagnostic test accuracy meta-analysis
Source: Arch Gynecol Obstet. 2022 Oct 31;308(2):435–51. doi: 10.1007/s00404-022-06812-w (PMC10293352; doi:10.1007/s00404-022-06812-w)
Supplement: Supplementary file 7 — Supplementary file7 Summary of Risk of Bias and Applicability Concerns according to QUADAS-2 score (DOCX 73 KB) [file 404_2022_6812_MOESM7_ESM.docx]

**Supplementary Table 2.** Summary of Risk of Bias and Applicability Concerns according to QUADAS-2 score

| **Study** | **RISK OF BIAS** | | | | **APPLICABILITY CONCERNS** | | |
| --- | --- | --- | --- | --- | --- | --- | --- |
|  | **PATIENT SELECTION** | **INDEX TEST** | **REFERENCE STANDARD** | **FLOW AND TIMING** | **PATIENT SELECTION** | **INDEX TEST** | **REFERENCE STANDARD** |
| Wagner et al. | ☺ | ☺ | ☺ | ☺ | ☺ | ☺ | ☺ |
| Wiechec et al. | ☺ | ? | ☺ | ☺ | ☺ | ? | ☺ |
| Burger et al. | ☹ | ☺ | ☺ | ☺ | ☺ | ☺ | ☺ |
| Mula et al. | ☺ | ☺ | ? | ☺ | ☺ | ☺ | ? |
| Turan et al. | ☺ | ☺ | ? | ☺ | ☺ | ☺ | ? |
| Yang et al. | ☺ | ☺ | ☺ | ☺ | ☺ | ☺ | ☺ |
| Borrell et al. | ☺ | ☺ | ☺ | ☺ | ? | ☺ | ☺ |
| Prats et al. | ☺ | ☺ | ? | ☺ | ☺ | ☺ | ☺ |
| Volpe et al. | ☺ | ☺ | ☺ | ☺ | ☺ | ☺ | ☺ |
| Chelemen et al. | ☺ | ☺ | ☺ | ☺ | ? | ☺ | ☺ |
| Clur et al. | ☹ | ? | ☺ | ☹ | ☺ | ☺ | ☺ |
| Timmerman et al. | ☺ | ☺ | ☺ | ☺ | ☺ | ☺ | ☺ |
| Martinez et al. | ☺ | ☺ | ☺ | ☺ | ☺ | ☺ | ☺ |
| Maiz et al. | ☺ | ? | ☺ | ☺ | ☺ | ☺ | ? |
| Maiz et al. (2) | ☺ | ? | ☺ | ☺ | ☺ | ☺ | ? |
| Toyama et al. | ☺ | ☺ | ☺ | ☺ | ☺ | ☺ | ☺ |
| Favre et al. | ☺ | ☺ | ☺ | ☺ | ☺ | ☺ | ☺ |
| Matias et al. | ☺ | ☺ | ? | ☺ | ☺ | ☺ | ☺ |
| Karadzov-Olic et al. | ☺ | ☺ | ☺ | ☺ | ☺ | ☺ | ☺ |
| Zoppi et al. | ☺ | ? | ☺ | ☹ | ☺ | ? | ☺ |
| Murta et al. | ☺ | ☺ | ☺ | ☹ | ☺ | ☺ | ☺ |
| Minella et al. | ☺ | ☺ | ☺ | ☺ | ☺ | ☺ | ☺ |

☺Low Risk ☹High Risk ? Unclear Risk
